# Proteomic Changes during B Cell Maturation: 2D-DIGE Approach

**DOI:** 10.1371/journal.pone.0077894

**Published:** 2013-10-29

**Authors:** Johanna Salonen, Gunilla Rönnholm, Nisse Kalkkinen, Mauno Vihinen

**Affiliations:** 1 Institute of Biomedical Technology, University of Tampere, Tampere, Finland; 2 BioMediTech, Tampere, Finland; 3 Research Unit, Tampere University Hospital, Tampere, Finland; 4 Institute of Biotechnology, University of Helsinki, Helsinki, Finland; 5 Department of Experimental Medical Science, Lund University, Lund, Sweden; Centro di Riferimento Oncologico, IRCCS National Cancer Institute, Italy

## Abstract

B cells play a pivotal role in adaptive immune system, since they maintain a delicate balance between recognition and clearance of foreign pathogens and tolerance to self. During maturation, B cells progress through a series of developmental stages defined by specific phenotypic surface markers and the rearrangement and expression of immunoglobulin (Ig) genes. To get insight into B cell proteome during the maturation pathway, we studied differential protein expression in eight human cell lines, which cover four distinctive developmental stages; early pre-B, pre-B, plasma cell and immature B cell upon anti-IgM stimulation. Our two-dimensional differential gel electrophoresis (2D-DIGE) and mass spectrometry based proteomic study indicates the involvement of large number of proteins with various functions. Notably, proteins related to cytoskeleton were relatively highly expressed in early pre-B and pre-B cells, whereas plasma cell proteome contained endoplasmic reticulum and Golgi system proteins. Our long time series analysis in anti-IgM stimulated Ramos B cells revealed the dynamic regulation of cytoskeleton organization, gene expression and metabolic pathways, among others. The findings are related to cellular processes in B cells and are discussed in relation to experimental information for the proteins and pathways they are involved in. Representative 2D-DIGE maps of different B cell maturation stages are available online at http://structure.bmc.lu.se/BcellProteome/.

## Introduction

B cells play a pivotal role in adaptive immune system, since they are needed to maintain a delicate balance between recognition and clearance of foreign pathogens and tolerance to self. During their maturation, B cells progress through a series of developmental stages defined by specific phenotypic surface markers and the rearrangement and expression of immunoglobulin (Ig) coding genes (for review see [1]). The maturation begins in bone marrow and foetal liver, and proceeds through ordered series of steps resulting in the release of immature B cells expressing surface IgM. These cells exit to periphery and migrate into spleen where they develop into mature B cells expressing surface IgM and IgD. Only a minority of the newly generated immature B cells enters the pool of mature B cells. The majority undergoes apoptosis by mechanisms, which prevent production of self-recognizing molecules. Once activated, following engagement of B cell antigen receptor (BCR) with antigen, mature B cells move to germinal centres in the lymphoid tissue and, with help of other cells, differentiate into antibody-secreting plasma or memory B cells.

The BCR structure and the signalling pathways following antigen binding are extensively studied (reviewed in [2], [3]). Briefly, the BCR consists of the membrane-bound Ig and depending on the stage of B cell differentiation, it is associated with a couple of transmembrane proteins, most notably Ig-α and –β [4]. BCR crosslinking via antigen engagement activates tyrosine-phosphorylation signalling pathways inside the cell. The phosphorylation of immunoreceptor tyrosine activation motifs in Ig-αβ by Lyn initiates the formation of an assembly of intracellular signalling proteins such as Syk, Bruton tyrosine kinase (Btk) and Vav. Together with adapter proteins, the assembly regulates down-stream signalling cascades including the mobilization of Ca^2+^ ions, the reorganization of cytoskeleton architecture, the activation of nuclear transcription factors and the induction of gene expression. Alterations to proteins and genes involved in the maturation cause primary immunodeficiencies (PIDs) [5]. These variations result in defective or aberrant B cell function and development. Either the development is impaired or the B cells generated fail to respond to T cell signals [6], [7]. Well over 150 PID-related genes and thousands of PID-causing variations have been identified [8]. PID patients have increased susceptibility to recurrent and persistent infections. Early antibiotic treatment and lifelong immunoglobulin replacement therapy is required for many patients.

What happens in the B cell proteome during maturation? Which proteins are co-expressed and what intracellular processes are activated or silenced? These questions are still largely unanswered despite extensive studies of individual genes and proteins (reviewed [9], [10]). To address the question, we analysed B cell proteomes all the way from early pre-B cell stage until terminally differentiated plasma cell stage with combined two-dimensional differential gel electrophoresis (2D-DIGE) and matrix-assisted laser desorption/ionization-time of flight-mass spectrometry (MALDI-TOF-MS). This approach allows proteome wide analysis of intracellular proteins.

Bioinformatic analysis of nearly 190 proteins revealed significant changes in functions of co-expressed proteins between maturation stages. Notably, cytoskeleton and ontogeny related proteins were moderately expressed in early pre-B/pre-B cell stages and silenced at immature B cell (IM-B) stage. The transition from pre-B to IM-B stage changed significantly the proteome profile, since proteins from a variety of functional gene ontology (GO) categories appeared. Our long time series analysis in anti-IgM stimulated Ramos B cells revealed the dynamic regulation of cytoskeleton, gene expression and metabolic pathways, among other cellular events. In plasma cell stage, the proteome contained endoplasmic reticulum (ER)/Golgi –system proteins needed for efficient antibody production. The results provide new proteome wide insights into B cell development and expand our previous transcriptomic and proteomic studies for anti-IgM stimulated Ramos B cells [11], [12], [13], [14], [15].

## Materials and Methods

### Cell Culture and Treatment

Nalm-1, Nalm-6, 380, 697, Ramos and U-266 cell lines were purchased from Leibniz Institute DSMZ-German Collection of Microorganisms and Cell Cultures. REH [16], Nalm-1 [17], Nalm-6 [18], 380 [19], 697 [20], Ramos [21] and U-266 [22] cells were cultured in RPMI 1640 medium, whereas RS4;11 [23] was cultured in α-MEM medium (for details about the cell lines see Table S1). All culture media were supplemented with 10–20% fetal bovine serum, 100 µg/ml streptomycin and 100 IU/ml penicillin. For BCR stimulation, Ramos cells were pelleted and suspended in RPMI culture medium supplemented with 25 mM HEPES (pH 7.4) and anti-human IgM (20 µg/ml Sigma I2386). Ramos cells were stimulated or left untreated for 1, 2, 4, 9, 12, 16, 24, 48, 72, 96 and 120 h. Stimulation and incubation were stopped by adding three volumes of ice-cold phosphate-buffered saline (PBS) containing protease inhibitors (Complete Protease Inhibitor Cocktail, Roche) and phosphatase inhibitors (1 mM sodium orthovanadate and 20 mM sodium fluoride). Two parallel biological samples of stimulated and control Ramos cells were prepared for each time point and three parallel samples were prepared for the other B cells. All cells were washed in ice-cold PBS containing inhibitors for proteases and phosphatases, pelleted and stored at −70°C until used.

### Protein Sample Preparation

B cell aliquots (about 5×10^7^ cells) were lysed in 200–650 µl of lysis buffer (8 M urea, 4% CHAPS, 30 mM Tris (pH 8.5) including protease inhibitors) for 30 minutes on ice. The protein concentration was determined using the DC Protein Assay as described by the manufacturer (Bio-Rad). An equal volume of ice-cold sample buffer (2 M thiourea, 7 M urea, 4% CHAPS, 30 mM Tris (pH 8.5) and protease inhibitors) was added on samples after protein concentration determination. Soluble proteins were separated by ultracentrifugation at 100 000×g for 35 min at 4°C.

### 2D-DIGE and Image Acquisition

Protein samples were labelled with CyDye DIGE Fluor minimal Cy2-, Cy3- and Cy5-dyes and prepared according to the manufacturer’s protocol (GE Healthcare). Briefly, 50 µg of each protein extract was separately labelled with 400 pmol of Cy3- and Cy5-dyes dissolved in DMF on ice for 30 min. A pooled internal standard sample was prepared by combining protein extracts from the eight different B cell lines and labelled with Cy2-dye as above. The labelling reactions were quenched by the addition of 1 µl of 10 µM L-lysine solution for 10 min. Prior to isoelectric focusing (IEF), labelled protein samples were added to an equal volume of DIGE sample buffer (7 M urea, 2 M thiourea, 4% CHAPS, 130 mM dithiotreitol, 2% IPG buffer 3–10 (GE Healthcare)) and left on ice for 10 min. The Cy2-, Cy3- and Cy5-dye labelled protein samples to be separated in the same gel were mixed and rehydration buffer (7 M urea, 2 M thiourea, 4% CHAPS, 13 mM dithiotreitol, 1% IPG buffer 3–10) was added to obtain a final volume of 450 µl. The proteins were separated by IEF and subsequently by 12% SDS-polyacrylamide gel electrophoresis as described previously [15]. The IEF was carried out using immobilized nonlinear pH gradient 3–10 gel strips (24 cm Immobiline DryStrip gels, GE Healthcare) and Ettan II IPGphor system (GE Healthcare) for a total of 40–50 kVh. The second dimension protein separation was carried out for 10 h at a constant wattage of 5 W per gel using Ettan DALTsix electrophoresis system (GE Healthcare).

For a statistical analysis of the B cell proteins, two parallel biological samples were prepared for each B cell line and time point using a dye-swap design to account for difference arising from dye-specific effects [24]. Altogether 31 2D-DIGE gels were prepared and analysed. For preparative gels, 180 µg of unlabelled protein per sample were applied into gel strips and 2D-gel electrophoresis (2D-GE) was performed as previously [15]. Proteins were post-stained with silver using a method compatible with the identification of proteins by mass spectrometry (MS) [25].

The Cy-dye labelled proteins in 2D-DIGE gels were visualized using a Typhoon Trio scanner (GE Healthcare). The Cy2-labelled protein map images were scanned using a 488 nm laser and an emission filter of 520 nm with a band-pass of 40 nm (520BP40), Cy3 images using a 532 nm laser with a 580BP30 filter and Cy5 images using a 633 nm laser with a 670BP30 filter.

### Protein Identification

Prior to identification, the silver stained proteins were in-gel alkylated and digested with trypsin (Sequencing Grade Modified Trypsin V5111, Promega) overnight at 37°C using a method adapted from Shevchenko et al. [26]. The recovered peptides were, after desalting using µC18 ZipTip (Millipore), subjected to MALDI-TOF-MS analysis. The proteins were identified using peptide mass fingerprinting (PMF) followed by database searches. For unreliable identifications, the protein identities were confirmed by fragment ion analysis. MALDI-TOF mass spectra for PMF and MALDI-TOF/TOF mass spectra for identification by fragment ion analysis were acquired using an Ultraflex TOF/TOF instrument (Bruker Daltonik), equipped with a 337 nm UV nitrogen laser, in a reflector ion mode using α-cyano-4-hydroxycinnamic acid as a matrix. The instrument was externally calibrated using a standard mixture of peptides from Bruker. Protein identification with the generated mass spectra data was performed using the Mascot Peptide Mass Fingerprint and MS/MS Ion Search programmes (Matrix Science). Mascot (V2.3.0.1) was used as a search engine against SwissProt (v SwissProt_2010_06.fasta) and NCBInr (20100816) databases with 64990 and 845292 mammalian sequences, respectively. Generated MS spectra were matched with a peptide mass tolerance of 100 ppm and fragment mass tolerance of 0.8 Da. One miss cleavage was permitted. Carbamidomethylation of Cys was selected as a fixed modification and oxidation of Met as a variable modification.

### 2D-DIGE Data Analysis

Intra- and inter-gel analyses for the 31 gels were carried out in DeCyder DIA (differential in-gel analysis) and BVA (biological variation analysis) modules, respectively (V6.5 GE Healthcare). After setting manual landmarks, gel-to-gel matching was performed in automatic mode. Protein spot matches were confirmed and mismatched spots were corrected manually. The normalized spot volume data of the 89 images (corresponding to 31 gels, since for certain gels only Cy3 or Cy5 image was used along with Cy2 standard image) were exported for further processing. The standardized volume ratios for the detected protein spots were calculated by dividing each normalized spot volume by its respective in-gel Cy2 internal standard volume and transformed logarithmically. Analysis of this data set consisted of three steps. First, the data was subjected to unsupervised cluster and scatter plot analyses for reproducibility evaluation of the biological replicates. Second, the averaged spot volumes were calculated for each protein and two-way hierarchical clustering and paired correlations were applied across the dataset. Third, to study proteins affected by the BCR stimulation, the 11 groups of anti-IgM stimulated Ramos B cells were compared to the corresponding control groups (1, 2, 4, 9, 12, 16, 24, 48, 72, 96 and 120 h), each group consisting of two biological replicates. The DeCyder BVA module was used to calculate average abundance changes and paired student’s t-test p-values for the variance of these ratios for each protein-pair across stimulated and control groups. Protein spots that exhibited at least a two-fold decrease or increase in relative protein volume and p-values ≤0.05 were considered as interesting and majority of them were identified by MS analysis. Identified proteins were matched to their corresponding UniProt entries using Blast. In the average linkage clustering, the uncentered correlation distance was used as a similarity metric. The protein abundance data was clustered using R (V2.1) (www.r-project.org) and Gene Cluster 2.11 [27] and the results were visualized using TreeView 1.60 (http://rana.lbl.gov/EisenSoftware.htm). The protein abundance profiles (1362–1827 protein spots across the cell lines) were investigated by a correlation matrix. Bivariate Pearson correlations were calculated using the SPSS (V17.0) software (Softonic).

### Bioinformatic Analysis for Differentially Expressed Proteins

To study B cell maturation stage related protein expression, the data was filtered to retain only those protein spots, whose volume changed two-fold or more with relation to internal standard in at least one cell line or time point. Out of the 836 proteins that passed the criteria, 174 were identified, grouped into hierarchical clusters and exposed to GO term [28] enrichment analysis followed by KEGG [29], BioCarta (http://www.biocarta.com) and Reactome [30] pathway analyses. Two-way hierarchical clustering algorithm based on the average linkage method was used to group the identified proteins. To follow the BCR–activation induced proteomic changes on time, the differences in relative protein volumes between anti-IgM stimulated and corresponding control Ramos B samples were calculated for the 69 identified proteins that passed the significance criteria (two-fold change in relative protein abundance with student’s t-test p-value ≤0.05). The proteins were clustered on the basis of the differential protein abundance and exposed to functional annotation analysis. The clustering results were visualized using TreeView.

To find significant shared GO terms within the clusters of co-expressed proteins, p-values were calculated using hypergeometric distribution. The calculations were performed using Gene Set Analysis Toolkit V2 [31] and the entire dataset (233 identified proteins encoded by 168 GO-annotated genes, Table S2) was set as a background for calculations. Criteria for the significance were that at least two proteins in a cluster share a GO term with p-value ≤0.05 adjusted by the Benjamini multiple test adjustment. If the same set of proteins were significantly enriched in both a parent and its child term, only the child was listed. The signalling pathway analyses were run using David V6.7 [32], and for the significance calculations human genome was set as a background. Exhaustive literature mining was performed to study biological roles of the proteins in B cell development.

## Results

**Figure 1:**
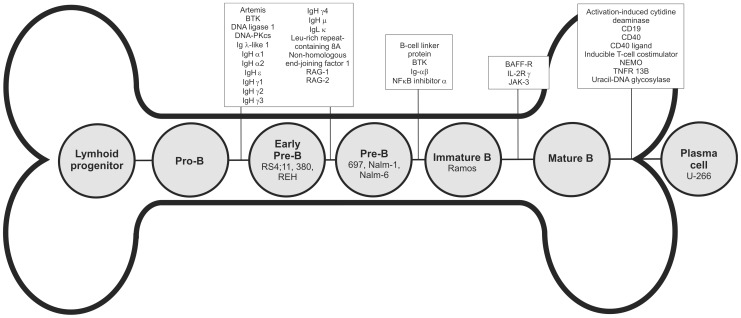
PID-related proteins in B cell maturation pathway. Defects in proteins and genes important for maturation cause PIDs and block the maturation on certain stage. The PID-related proteins and stages where diseases affect the development are shown on top. The cell lines (RS4;11, 380, REH, 697, Nalm-1, Nalm-6, Ramos and U-266) are marked inside the corresponding cells representing their normal B cell counterparts.

Proteome wide changes during the maturation of B cells from early pre-B cell stage until plasma cell stage were investigated using 2D-DIGE and MALDI-TOF-MS based proteomics. This approach makes use of the Cy-dye technology in the analysis of differential protein expression permitting greater confidence compared to the traditional 2D-GE. The proteomic changes were investigated across eight human cell lines, which together cover four distinctive differentiation stages in the maturation pathway (see Figure 1). Further, to study proteins affected by BCR activation, the proteome profiles of anti-IgM stimulated and control Ramos B cells were compared in a time series 1–120 h. Determination of protein expression patterns at each stage provides information about regulation of cellular processes, such as Ig chain production, during these stages.

### B Cell Proteomes

**Figure 2:**
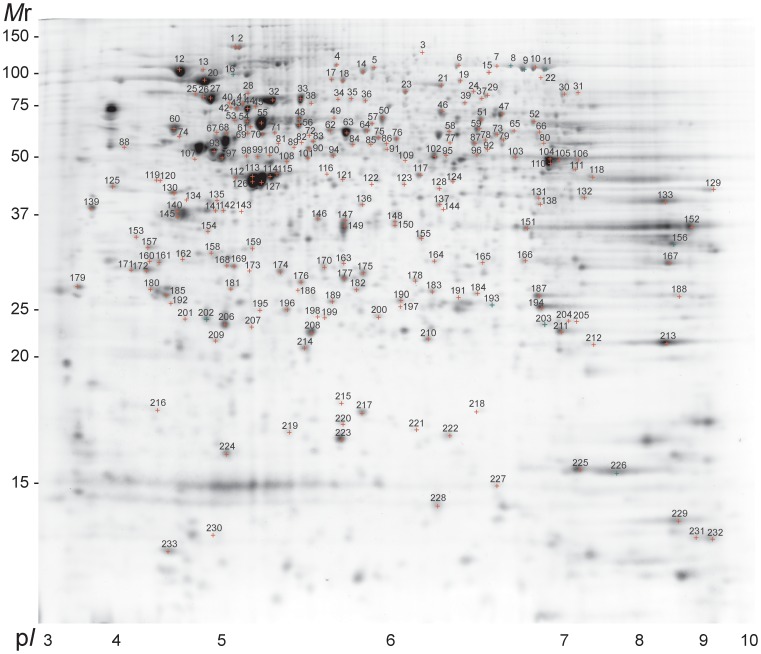
Reference 2D-DIGE map of Cy2-labelled internal standard. The 233 identified proteins are numbered in the gel and listed in Table S2.

DeCyder DIA analysis of the 31 gels resulted in detection and quantitation of 1951–2467 protein spots per gel. A reference gel of Cy2-labelled internal standard sample with 2063 spots was used as master gel for DeCyder BVA analysis (Figure 2). BVA analysis resulted in matching of 1213–1702 spots across the gels and matching of 1599–1827 spots (78–89%) across the cell lines. The reproducibility of the protein abundance profiles of biological replicates was evaluated by scatter plot and unsupervised analyses. Two independent 2D-DIGE separations of each protein extract separately labelled with Cy3- and Cy5-dyes were performed, and the correlation of relative spot intensity (Cy3/Cy2 vs. Cy5/Cy2) was examined. The Pearson correlation coefficient for all pairs of biological replicates was 0.6–0.8.

### B Cell Proteome Database

Interactive B cell proteome database contains representative 2D-DIGE maps of each maturation stage and information about the protein identifications. The 233 protein sample spots (SSP) are numbered in the gel image (Figure 2) and listed in Table S2 with Mascot scores to show the reliability of the identifications (p<0.05). The database contains 125 new identifications based on PMF or by combined PMF and fragment ion analysis in addition to previous identifications from 2D-GE Ramos dataset [15]. The peptides identified from different proteins by fragment ion analysis are listed in Table S3. The database is available online at http://structure.bmc.lu.se/BcellProteome/.

### B Cell Proteomes Cluster According to their Maturation Stage

**Figure 3:**
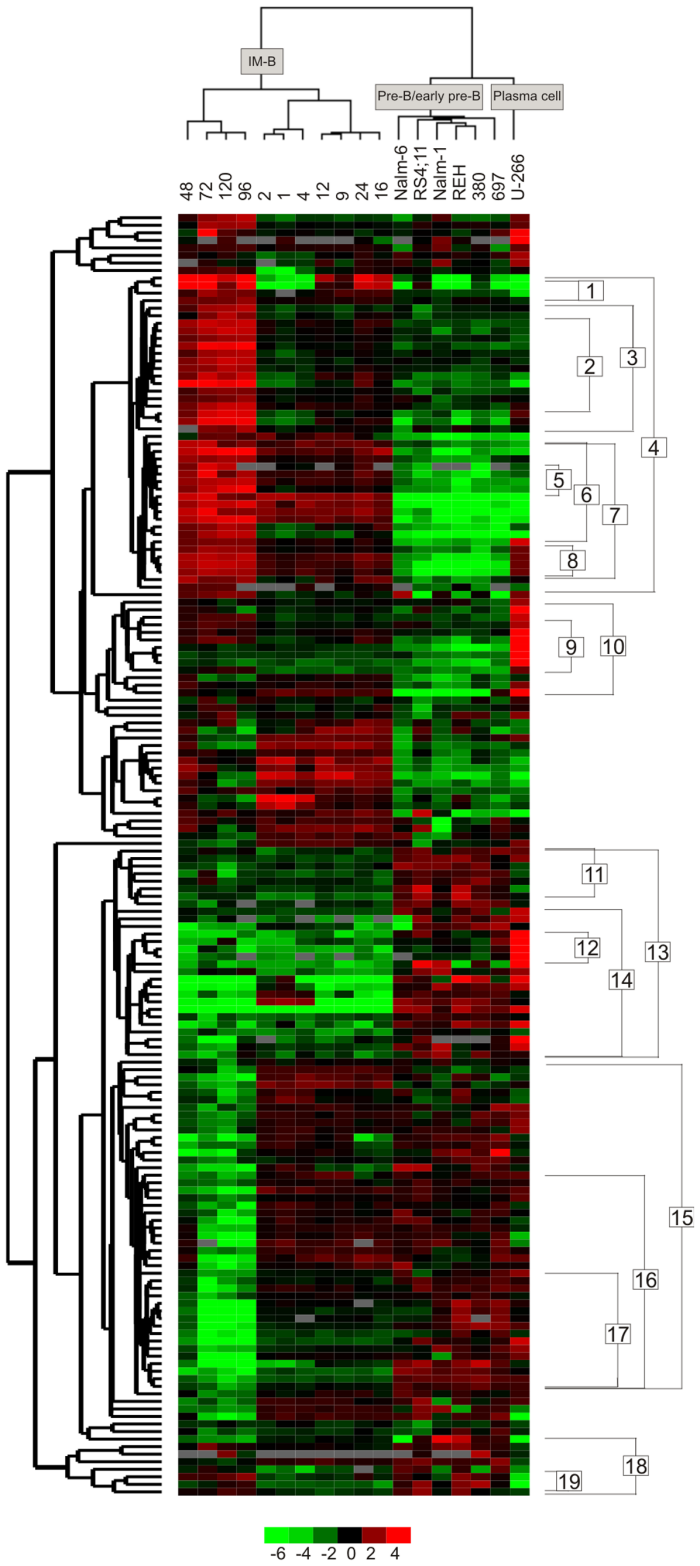
Unsupervised clustering of B cell samples on the basis of protein profiles. IM-B Ramos samples clustered according to time after anti-IgM stimulation (1–120 h) and were distinctive from early pre-B (RS4;11, 380 and REH), pre-B (697, Nalm-1 and Nalm-6) and plasma cell U-266 samples. The degree of similarity in profiles is shown by colour (base-two logarithmical scale below the figure). Red and green indicate high and low levels of protein expression in relative to internal standard. The average protein abundance of the 175 identified proteins (labelled with Cy3 and Cy5 dyes) is calculated relative to internal standard (labelled with Cy2 dye). Cluster numbers (1–19) are shown to the right and refer to those in Table 1, Table 2, Table 3, Table S2 and Table S4.

**Figure 4:**
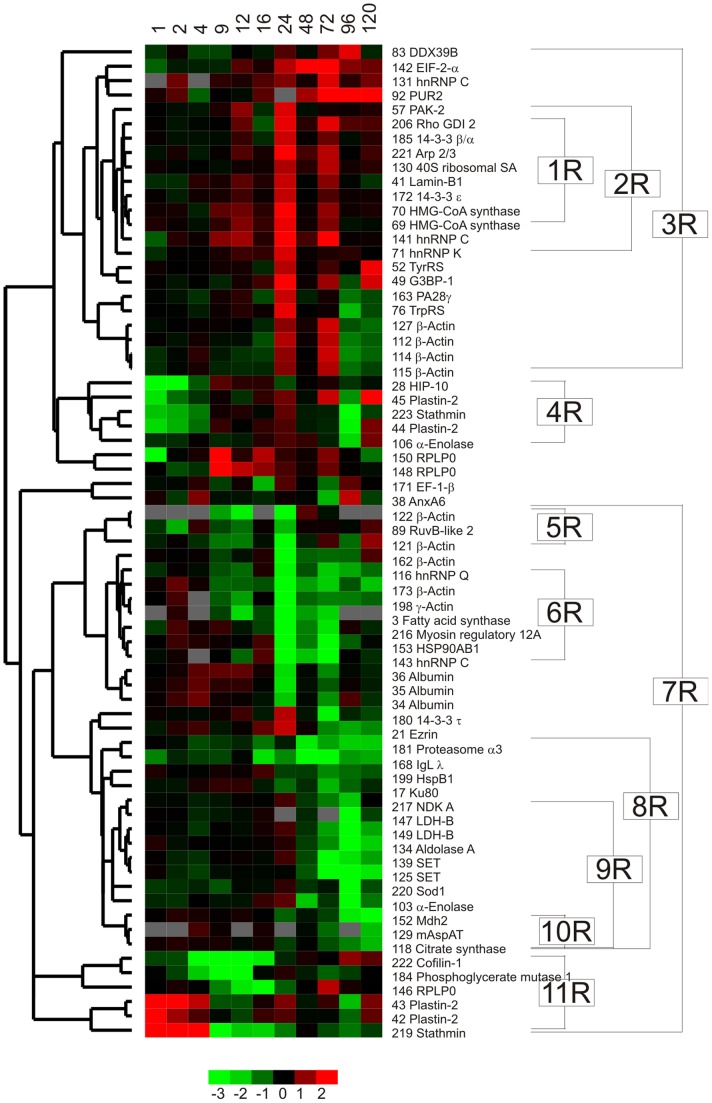
Unsupervised clustering of proteins affected by anti-IgM stimulation. The heat map shows the comparison between anti-IgM stimulated and control Ramos samples during the time course 1–120 h. Protein abundance difference is calculated between stimulated and corresponding control samples for the 69 identified proteins, which passed the significance criteria (student’s t-test p≤0.05). Red and green colours indicate up- and down-regulation in response to anti-IgM stimulation (base-two logarithmical scale below the figure). Cluster numbers (1R-11R), short protein names and SSP are shown to the right and refer to those in Table 1, Table 2, Table 3, Table S2 and Table S4.

In unsupervised analyses, the datasets clustered into maturation stage related groups with distinctive pattern of Ramos B samples (Figure 3, Figure S1 and Figure S2). The IM-B Ramos cells clustered according to time and were distinct from the other cells. The plasma cell U-266 distinguished from the early pre-B (380, REH and RS4;11) and pre-B cells (697, Nalm-1 and Nalm-6), which showed rather homogenous proteome profiles. The result reflects the biological similarity of early pre-B and pre-B lineages (Table S1) and indicates that early during the differentiation pathway, the proteomes are still close to each other. The correlation matrix suggests dependencies between protein expressions of consecutive stages, with strongest positive correlation between successive Ramos B subsets and between early pre-B and pre-B cells (see Figure S1 right). Interestingly, Ramos B cells at late time points and early pre-B/pre-B cells have weak negative correlation. The cluster dendrogram shows the high similarity between stimulated and corresponding control Ramos B proteomes indicating minor anti-IgM stimulation induced changes, except at 24 h and at the end of the time course (96–120 h) (see Figure S2). Those changes in protein expression according to time are shown in detail in Figure 4.

### Enriched GO Terms and Signalling Pathways

**Figure 5:**
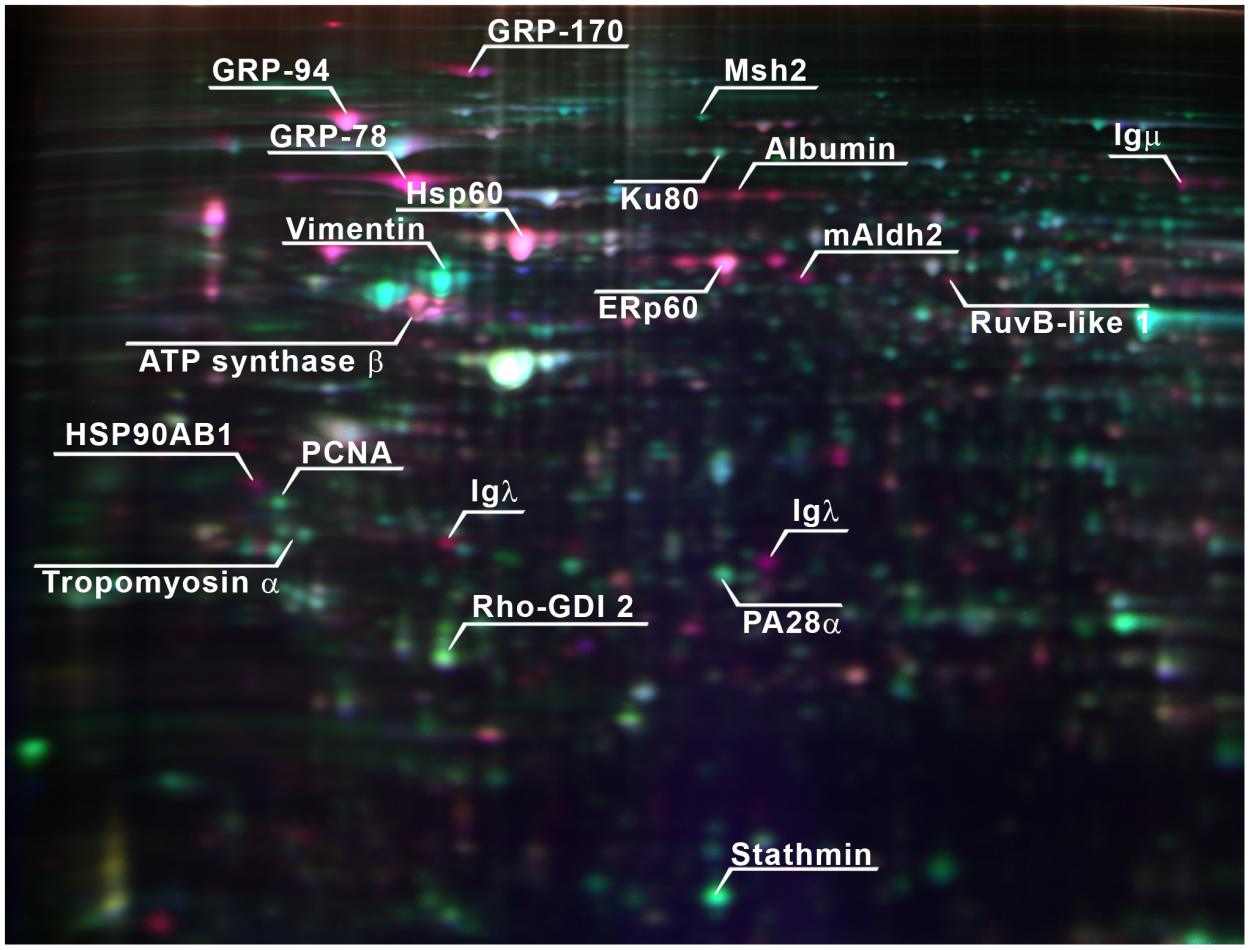
Differences in proteome profiles between IM-B and early pre-B cell stages. Early pre-B 380 and IM-B Ramos (120 h time point) proteome samples were labelled with Cy5 and Cy3 dyes, respectively, and separated in a 2D-DIGE gel (nonlinear pH gradient 3–10) with internal standard sample labelled with Cy2 dye. Triple image overlay shows proteins highly expressed in Ramos (red) and 380 (green) samples in relative to internal standard sample (blue). Protein identities are indicated for those proteins with major changes in expression profile. IgM chains, ER and membrane bound vesicle proteins were highly expressed in Ramos cells, whereas cytoskeleton and immunity related proteins were expressed in 380 cells.

Filtering of the data and MS analysis resulted in identification of 188 proteins encoded by 131 GO-annotated genes. Of these identified proteins, 174 were clustered based on the relative abundance profiles in different B cell lines (Figure 3), whereas 69 were clustered based on the abundance differences between anti-IgM stimulated and control Ramos B cells in a time series (Figure 4). Thus, certain proteins are present in both datasets indicating differential protein expression upon anti-IgM stimulation as well as differential expression between certain maturation stages (see Table S2). Significant changes in functions of co-expressed proteins were revealed with greatest differences between early pre-B/pre-B and IM-B cells (see Figure 5). Bioinformatic analysis exposed sheared Biological process, Molecular function and Cellular component GO terms, as well as signalling pathways in the clusters of co-expressed proteins (Table 1, Table 2, Table 3 and Table S4, respectively). The unsupervised clustering revealed progressive changes in expressed proteins upon anti-IgM stimulation in Ramos B cells (Figure 4). In the early stage membrane-cytoskeleton linked proteins were regulated followed by the expression of many downstream components, like FAS pathway, rearrangement of cytoskeleton and initiation of gene expression in 24 hour. The same sets of proteins were re-regulated after 72 h reflecting the phase in cell division cycle. In the end of the time course, metabolic proteins were down-regulated indicating decreased energy requirement and cell death in Ramos B cells.

**Table 1:** Biological process GO terms and their p-values for co-expressed proteins.

GO ID	GO term	Protein (SSP)a)	P adj.b)	Clusterc)
16570	Histone modification	Prohibitin (176) ruvB-like 1 (77, 87) ruvB-like 2 (89, 90)	0.0536	2
16569	Covalent chromatinmodification	Prohibitin (176) ruvB-like 1 (77, 87) ruvB-like 2 (89, 90)	0.0536	2
6119	Oxidative phosphorylation	ATP synthase β (93) dihydrolipoyl dehydrogenase(66) cytochrome b-c1 (101)	0.0536	2
16568	Chromatin modification	CBX2 (80) prohibitin (176) ruvB-like 1 (77, 87) ruvB-like 2 (89, 90)	0.0306	3
9117	Nucleotide metabolic	Aldolase A (134) dUTPase (215) IDH3A (136) LDH-B (147, 149) malatedehydrogenase 1 (Mdh1) (151) NDK A (217) phosphoglycerate mutase 1 (187)PURH (47) transaldolase (137) triosephosphate isomerase (193, 194)	0.0021	15
19362	Pyridine nucleotidemetabolic	IDH3A (136) LDH-B (147, 149) Mdh1 (151) phosphoglycerate mutase1 (187) transaldolase (137) triosephosphate isomerase (193, 194)	0.0055	15
6007	Glucose catabolic	Aldolase A (134) aldolase C (132) α-enolase (95, 104, 105, 106, 110)LDH-B (147, 149) Mdh1 (151) phosphoglycerate mutase 1 (187)transaldolase (137) triosephosphate isomerase (193, 194)	0.0055	15
46496	Nicotinamide nucleotidemetabolic	IDH3A (136) LDH-B (147, 149) Mdh1 (151), phosphoglycerate mutase1 (187) transaldolase (137) triosephosphate isomerase (193, 194)	0.0055	15
6096	Glycolysis	Aldolase A (134) aldolase C (132) α-enolase (95, 104, 105, 106, 110)LDH-B (147, 149) Mdh1 (151) phosphoglycerate mutase 1 (187)triosephosphate isomerase (193, 194)	0.0093	15
8360	Regulation of cell shape	Aldolase A (134) coronin-1A (59) ezrin (21)	0.0499	15
6098	Pentose-phosphate shunt	phosphoglycerate mutase 1 (187) transaldolase (137)triosephosphate isomerase (193, 194)	0.0138	16
6913	Nucleocytoplasmic transport	DDX39B (82) eIF-5A-1 (224) Prdx-1 (212)	0.0467	18
6406	mRNA export from nucleus	DDX39B (82) eIF-5A-1 (224)	0.0467	18
6417	Regulation of translation	eIF-5A-1 (224) HspB1 (197)	0.0534	18
6917	Induction of apoptosis	eIF-5A-1 (224) Prdx-1 (212)	0.0534	18
6955	Immune response	Rho GDI 2 (206) Prdx-1 (212)	0.0378	19
35308	Negative regulation of protein aminoacid dephosphorylation	14-3-3 β/α (185) 14-3-3 ε (172)	0.021	1R
44248	Cellular catabolic	Aldolase A (134) α-enolase (103) citrate synthase (118)mAspAT (129) LDH-B (147, 149) Mdh2 (152)proteasome α3 (181) Sod1 (220)	0.03	8R
44262	Cellular carbohydrate metabolic	Aldolase A (134) citrate synthase (118)α-enolase (103) LDH-B (147, 149) Mdh2 (152)	0.03	8R
6107	Oxaloacetate metabolic	Citrate synthase (118) mAspAT (129) malate dehydrogenase (152)	0.03	8R
6096	Glycolysis	Aldolase A (134) α-enolase (103) LDH-B (147, 149) Mdh2 (152)	0.03	8R
30097	Hemopoiesis	NDK A (217) Sod1 (220) Ku80 (17)	0.0317	8R
50793	Regulation of developmental process	Aldolase A (134) Ku80 (17) NDK A (217) Sod1 (220)	0.0347	8R
46496	Nicotinamide nucleotide metabolic	Aldolase A (134) LDH-B (147, 149) Mdh2 (152) NDK A (217)	0.0374	8R
48523	Negative regulation ofcellular process	α-Enolase (103) HspB1 (199) NDK A(217) SET (125, 139) Sod1 (220) Ku80 (17)	0.045	8R
8152	Metabolic	Aldolase A (134) citrate synthase (118) α-enolase (103) HspB1 (199)Ku80 (17) LDH-B (147, 149) mAspAT (129) Mdh2 (152), NDK A (217)proteasome α3 (181) SET (125, 139) Sod1 (220)	0.0482	8R
7010	Cytoskeleton organization	Cofilin-1 (222) plastin-2 (42, 43) stathmin (219)	0.0487	11R
51261	Protein depolymerization	Cofilin-1 (222) stathmin (219)	0.0513	11R

a) SSP referring to those numbers in Figure 2 and short protein names referring to those proteins listed in Table S2.

b) P-value adjusted by the Benjamini multiple test adjustment.

c) Cluster numbers referring to those in Figure 3 and Figure 4.

**Table 2:** Molecular function GO terms and their p-values for co-expressed proteins.

GO ID	GO term	Protein (SSP)a)	P adj.b)	Clusterc)
3723	RNA binding	hnRNP C1/C2 (131) 60S ribosomal SA (148, 150)	0.039	1
15075	Ion transmembrane transporter activity	ATP synthase β (93) cytochrome b-c1 (101) VDAC-1 (167)	0.0169	2
15078	Hydrogen-ion transmembrane transporter activity	ATP synthase β (93) cytochrome b-c1 (101)	0.0305	2
5544	Calcium-dependent phospholipids binding	AnxA5 (58) AnxA6 (38)	0.0091	12
19901	Protein kinase binding	β-Actin (115) phosphoglycerate mutase 1 (187) vimentin (67, 68)	0.0352	14
5200	Structural constituent of cytoskeleton	β-Actin (115) moesin (24) vimentin (67, 68)	0.0447	14
50815	Phosphoserine binding	14-3-3 β/α (185) 14-3-3 ε (172)	0.0134	1R
42826	Histone deacetylase binding	14-3-3 β/α (185) 14-3-3 ε (172)	0.0134	1R
3723	RNA binding	eIF-2-α (142) DDX39B (83) G3BP-1 (49) hnRNPC1/C2 (141), hnRNP K (71) TyrRS (52)	0.032	3R
8092	Cytoskeletal protein binding	Cofilin-1 (222) plastin-2 (42, 43) stathmin (219)	0.035	11R

a) SSP referring to those numbers in Figure 2 and short protein names referring to those proteins listed in Table S2.

b) P-value adjusted by the Benjamini multiple test adjustment.

c) Cluster numbers referring to those in Figure 3 and Figure 4.

**Table 3:** Cellular component GO terms and their p-values for co-expressed proteins.

GO ID	GO term	Protein (SSP)a)	P adj.b)	Clusterc)
5743	Mitochondrial innermembrane	ATP synthase β (93) cytochrome b-c1 (101) Hsp60 (54, 55),prohibitin (176) VDAC-1 (167)	0.0018	2
44429	Mitochondrial part	ATP synthase β (93) cytochrome b-c1 (101) dihydrolipoyldehydrogenase (66) Hsp60 (54, 55) prohibitin (176) VDAC-1 (167)	0.0059	2
35267	NuA4 HAT complex	RuvB-like 1 (77, 87) ruvB-like 2 (89, 90)	0.0441	2
42645	Mitochondrial nucleoid	ATP synthase β (93) VDAC-1 (167)	0.0441	2
5759	Mitochondrial matrix	ATP synthase β (93) dihydrolipoyl dehydrogenase(66) Hsp60 (54, 55) VDAC-1 (167)	0.0441	2
30141	Secretory granule	Albumin (35, 36) dihydrolipoyl dehydrogenase (66) Hsp60 (54, 55)	0.015	3
5743	Mitochondrial innermembrane	ATP synthase β (93) cytochrome b-c1 complex 1 (101)Hsp60 (55, 54) mAspAT (129) prohibitin (176) VDAC-1 (167)	0.0187	4
44429	Mitochondrial part	ATP synthase β (93) cytochrome b-c1 complex 1 (101) dihydrolipoyldehydrogenase (66) Hsp60 (54, 55) mitochondrial aldehyde dehydrogenase 2(mAldh2) (75) mAspAT (129) prohibitin (176) VDAC-1 (167)	0.0302	4
31974	Membrane-enclosedlumen	Albumin (35, 36) mAldh2 (75) ATP synthase β (93) cytochromeb-c1complex 1 (101) dihydrolipoyl dehydrogenase (66) GRP-78 (25, 26, 27)GRP-94 (12) GRP-170 (1, 2) Hsp60 (54, 55) mAspAT (129), PDI (60) prohibitin(176) ruvB-like 1 (77, 87) ruvB-like 2 (89, 90) VDAC-1 (167)	0.032	4
5788	ER lumen	ERp60 (62, 63) GRP-170 (1, 2)	0.0407	5
5788	ER lumen	ERp60 (62, 63, 64) GRP-78 (25, 26, 27) GRP-94 (12) GRP-170 (1, 2) PDI (60)	0.0004	7
42470	Melanosome	ERp60 (62, 64, 63) GRP-78 (25, 26, 27) GRP-94 (12) HSP90AB1 (153) PDI (60)	0.0168	7
5788	ER lumen	GRP-78 (25, 26, 27) GRP-94 (12) PDI (60)	0.0011	8
5789	ER membrane	GRP-78 (25, 26, 27) GRP-94 (12)	0.0021	8
42470	Melanosome	GRP-78 (25, 26, 27) GRP-94 (12) PDI (60)	0.0046	8
5793	ER-Golgi intermediatecompartment	GRP-78 (25, 26, 27) PDI (60)	0.0059	8
42598	Vesicular fraction	GRP-94 (12) PDI (60)	0.0105	8
5783	ER	Neutral α-glucosidase AB (5, 14) ERp5 (97) SET (125) TXNDC5 (99, 100, 108)	0.0074	10
30529	Ribonucleoproteincomplex	β-Actin (115, 114, 112, 127) eIF-2-α (142) hnRNP C1/C2 (131, 141, 143)hnRNP K (71) hnRNP Q (116) DDX39B (83) 40S ribosomal protein SA (130)	0.0222	3R, 6R
3167	NuA4 HAT complex	β-Actin (121, 122) ruvB-like 2 (89)	0.0017	5R

a) SSP referring to those numbers in Figure 2 and short protein names referring to those proteins listed in Table S2.

b) P-value adjusted by the Benjamini multiple test adjustment.

c) Cluster numbers referring to those in Figure 3 and Figure 4.

## Discussion

Numerous previous studies have revealed the importance and function of either single or few proteins during B cell maturation or at certain maturation stages [33], [34], [35], [36], [37], [38]. Here, we performed an extensive proteomic analysis utilizing eight human B cell lines. These cell lines are widely used and resemble their normal B cell counterparts, but they do also have certain characteristics due to the cell line selection (Table S1). Our results indicate the involvement of 188 proteins encoded by 131 different genes. Among them 19 genes/proteins (15%) are already known to be involved in B cell differentiation.

### Immunity and Cytoskeleton Associated Proteins are Regulated during Maturation

Proteins highly or moderately expressed in early pre-B and pre-B stages clustered into two different clusters (numbered 18 and 13 in Figure 3). The first cluster accommodates six proteins (eukaryotic translation initiation factor (eIF)-5A-1, Rho-GDP-dissociation inhibitor 2 (Rho-GDI2), peroxiredoxin-1 (Prdx-1), RNA helicase DDX39B, heat-shock protein β-1 (HspΒ1) and lamin-B1) with various functions and decreased abundance after pre-B stage (immune response GO:6955, nucleocytoplasmic transport GO:6913, regulation of translation GO:6417 and induction of apoptosis GO:6917 in Table 1, and cluster 18 in Figure 3). The expression of Rho-GDI2 (see Figure 5) and Prdx-1 reached a minimum in plasma cell stage. Interestingly, Prdx-1 (SSP 212 and 213 in Figure 2) and RNA helicase DDX39B (SSP 82 and 83 in Figure 2) clustered not only based on their protein expression, but also based on their isoelectric point (pI) values indicating post-translational modification (PTM) specific co-expression. The second cluster accommodates seven cytoskeleton linked proteins, namely cofilin-1, profilin-1, tropomyosin α-3 and α-4, vimentin, β-actin and moesin (GO:5200 in Table 2, and cluster 13 in Figure 3), as well as additional proteins of interest. Some of these proteins had decreasing expression profile after pre-B stage, like tropomyosin α-4 and profilin-1, whereas the others were re-expressed in plasma cell stage, like tropomyosin α-3 and vimentin (Figure 5). In line with this, vimentin is expressed in many B cell types except Burkitt lymphoma [39]. Annexin A5 (AnxA5) had an unique expression profile, since it was highly expressed in early pre-B cells, silenced in pre-B and IM-B phenotypes and re-expressed in plasma cell type. Annexins bind to Ca^2+^-ions and the co-expression of AnxA5/A6 with cytoskeleton linked proteins (cluster 13 in Figure 3) might be related to Ca^2+^-signalling and regulation of membrane-cytoskeleton organization in early pre-B cells. Variations in cytoskeleton regulators are associated with the development of antibody deficiency syndromes in humans indicating their importance during B cell differentiation [40]. Also in developing chicken B cells, the dynamic regulation of cytoskeleton linked proteins was shown recently [35].

Although the proteomes of early pre-B and pre-B stages were highly similar, single proteins like ezrin, moesin, and ubiquitin-like domain-containing CTD phosphatase 1 (UBLCP1) were differentially expressed between early pre-B and pre-B groups. Moesin was highly expressed in early pre-B stage (380, REH and RS4;11), whereas ezrin was not expressed until in pre-B stage (697 and Nalm-6). Interestingly, ezrin was also regulated in 24 h upon anti-IgM stimulation (discussed in section: Proteins in different signalling pathways act together at 24 h), whereas ezrin and moesin were regulated early upon anti-IgM stimulation in Ramos B cells [37]. Ezrin and moesin are members of the ezrin-radixin-moesin family, members of which are involved in signal transduction to the actin cytoskeleton through Rho GTPases [41] whereby there is a negative feedback between activation of moesin and activity of the Rho pathway [42]. UBLCP1 had interesting yet unique protein abundance profile with decreased abundance in pre-B stage (697, Nalm-1 and Nalm-6) in relative to other developmental stages with moderate expression. UBLCP1 dephosphorylates nuclear 26S proteasomes, thereby decreasing their proteolytic activity [43]. The role of ubiquitin-proteasome system in respect to immune system is reviewed in [44].

In addition, we identified several proteins known to be regulated during B or T cell maturation, like DNA mismatch repair protein Msh2, inosine-5′-monophosphate dehydrogenase 2 (IMPD2), proliferating cell nuclear antigen (PCNA), Ku80, Ras GTPase-activating protein-binding protein 1 (G3BP-1), superoxide dismutase 1 (Sod1) and HspΒ1. The expression of these proteins reflects many known aspects of B cell ontogeny but also reveals many novel features. Msh2 and PCNA take part in DNA mismatch repair (GO:6298) and their protein abundance decreased after pre-B stage (see Figure 5 and cluster 13 in Figure 3). The expression of Msh2 and PCNA reached a minimum in Nalm-6 and 697 cells, respectively. Nalm-6 represents the most developed pre-B cell in the basis of its membrane μ expression [18]. Msh2 has a special role in B cell differentiation, since it is needed in somatic hypermutation of Ig genes, which results in amino acid changes in the rearranged V regions of Ig [45]. In line with our results, PCNA was highly expressed in mice pre-B cells [46]. Ku80 and G3BP-1 were moderately expressed in 380 and REH or Nalm-1 cells. G3BP-1 takes part in Ras signalling, which is essential for pre-B cell development. The expression of dominant negative Ras blocks development at the pro-B cell stage [47], whereas the expression of constitutively active Ras induces developmental progression and IgL recombination [48]. Ku is a component of DNA non-homologous end joining machinery, which joins the DNA ends and completes the V(D)J recombination event [49]. IMPD2 and Sod1 were highly expressed in pre-B and plasma cells compared to Ramos IM-B cells (clusters 11 and 17, respectively, in Fig. 3). The importance of Sod1 and IMPD2 has been shown in T cells [50], [51], but not previously in B cells. The protein abundance pattern of HspΒ1 (cluster 18 Figure 3) was in accordance with the maturation stage of normal B-lineage cells [52].

### Transition from Pre-B to IM-B Cell Stage: Striking Changes in the Proteome Profile

The transition from pre-B to IM-B stage changed significantly the proteome profile, since proteins in six additional GO categories appeared. RNA binding (GO:3723 in Table 2), chromatin modification (GO:16568 in Table 1), mitochondrial and secretory granule proteins (GO:44429 and GO:30141 in Table 3) were selectively expressed in IM-B stage (clusters 1 and 3 in Figure 3), whereas the expression of ER and melanosome proteins (GO:5788 and GO:42470 in Table 3) continued or increased until plasma cell stage (cluster 8 in Figure 3). The co-expression of IgM, non-specific lipid-transfer protein, protein disulfide-isomerase A3 (ERp60), hypoxia up-regulated protein 1 (GRP-170) (GO:5788 and cluster 5 in Table 3) and cytoplasmic membrane bound vesicle proteins; Hsp60 and HSP90AB1, indicate active IgM chain production and transport in Ramos cells (see Figure 5, and cluster 6 in Figure 3). The relatively high expression of chromobox protein homolog 2 (CBX2), ruvB-like 1/2 and prohibitin indicate regulation of transcription via chromatin structure modification (GO:16568 in Table 1, and cluster 3 in Figure 3). Noticeably, some of these proteins have special functions in developing B cells, like prohibitin, which interacts selectively with IgM [53]. RuvB-like 1/2 possesses single-stranded DNA-stimulated ATPase and ATP-dependent DNA helicase activities and is a component of the NuA4 histone acetyltransferase (HAT) complex. NuA4 HAT induces transcriptional activation and open chromatin structure by acetylating nucleosomal histones [54]. Interestingly, the more acidic isoforms of ruvB-like 1 and −2 (see Figure 5, and SSP 77 and 89 in Figure 2) were expressed exclusively in Ramos B cells indicating PTM specific regulation. CBX2 is a component of the Polycomb group multiprotein PRC1-like complex, which maintains transcriptionally repressive state of many genes throughout lymphopoiesis [55]. The reduction of enzymes involved in nucleotide metabolism and glucose catabolism (GO:9117, GO:6007, cluster 15 in Table 1) indicates reduced metabolism in Ramos cells at 72–120 h compared to other cell stages.

### Plasma Cell is Specialized in the Expression of ER and Ig Secretory Machine Proteins

The transition from IM-B to plasma cell stage narrowed the repertoire in functions of co-expressed proteins. When Ramos cells express proteins from a variety of functional GO categories, the U-266 cells are specialized in ER/Golgi system related protein expression. Among the ER resident proteins, the expression of ERp5, ERp46 and TXNDC5 continued from IM-B stage to plasma cell stage, whereas the expression of ERp60 and GRP-170 was silenced in plasma cell stage (cluster 9 and 5, respectively, in Figure 3). The overlapping but diverse expression of ER and folding enzymes indicates different mechanism for Ig chain processing and transport in U-266 and Ramos cells. These differences likely reflect the different functions and phenotypes of the IM-B and plasma cells. Ramos B cells express IgM on their cell surface and secrete it in small amounts, whereas U-266 cells secrete vast amounts of IgE but lack membrane Ig [22], [56]. Further, gene-expression programs of antigen activated B cells are different from those of plasma cells (reviewed in [9]).

Proteins elevated in plasma cell clustered into three different clusters based on their expression patterns across the maturation pathway. The first cluster (number 8 in Figure 3) contains ER/Golgi proteins (GO: 5788, GO: 5789, GO: 5793 in Table 3) and membrane bound vesicle proteins (GO:42470, GO:42598 in Table 3), which were not expressed until in IM-B stage. For example, the expression of 78 kDa glucose-regulated protein (GRP-78) and endoplasmin (GRP-94) was elevated in Ramos B cells at 48–120 h (see Figure 5), whereas the expression of protein disulfide isomerase (PDI) reached a maximum in U-266 cells. In line with this, the requirement of efficient Ig assembly and disulfide bond isomerization increases during the maturation, as confirmed by observations of others [57], [58]. The second cluster accommodates ER proteins (GO:5783 and cluster 9 in Figure 3 and Table 3) and five enzymes with peptidase or isomerase activity; peptidyl-prolyl isomerase, cytosolic non-specific dipeptidase, cytosol aminopeptidase, proteasome subunit α type 7 and ubiquitin carboxyl-terminal hydrolase isozyme L1. These proteins were not highly expressed until in terminally differentiated plasma cell stage, as shown previously in mouse B cells [38], [58]. Interestingly, synaptic vesicle membrane protein VAT-1 clustered in the same group indicating a possible new function of VAT-1 in U-266 plasma cells. The third cluster (number 12 in Figure 3) contains five proteins with U- or J-shaped expression profiles, namely AnxA5/6, γ-enolase, galectin-1 (GAL-1) and melanoma-associated antigen 4 (MAGE 4). AnxA5/6 had relatively moderate expression in early pre-B/pre-B cells, negligible in IM-B and high expression in plasma cell stage. AnxA5 and A6 interact with phospholipids in the presence of calcium (GO:5544 in Table 2) and dock onto membrane structures such as Golgi, ER and vesicles [59], [60]. Since these organelles are elevated in plasma cells [61], it is not surprising that AnxA5/6 expression was high in U-266 cells. Like AnxA5/6, γ-enolase and VAT-1 are calcium dependent, and their high expression might be related to ER, where calcium concentration is high. GAL-1 promotes Ig production and plasma cell differentiation [62]. In line with our result, GAL-1 expression is regulated during B cell development [63]. MAGE 4 may have a role in embryonic development and tumour progression, but its function is not known indicating its potential new function in U-266 cells.

### Regulation of Signalling Pathways during the Maturation

The correlation matrix suggests reverse expression patterns between early pre-B/pre-B and activated IM-B Ramos cells at late time points 48–96 h (see Figure S1 right). The signalling pathway analysis revealed many proteins with reverse expression between IM-B and early pre-B/pre-B cells and the pathways they are involved in (clusters 4, 14, and 15 in Table S4 and Figure 3). Some of these pathways reflect common cellular metabolic processes such as DNA replication, cell cycle control and metabolism of carbohydrate and amino acids (Reactome:383, Reactome:1538, Reactome:474 and Reactome:13, respectively, Table S4). The BCR stimulation in Ramos cells strengthened the down-regulation of many metabolic enzymes compared to resting Ramos cells (see Reactome:474 and clusters 7R, 4, 14 and 15 in Table S4). The low expression of these proteins in Ramos cells may simply reflect the cycling status. Interestingly, Btk was co-expressed with cell cycle control and DNA replication proteins. For proper BCR signalling, ordered activation of protein tyrosine kinases (PTK) Lyn, Syk and Btk is required. Deficiencies in these PTKs cause defective or aberrant B cell function and development (see Figure 1) [64], [65], [66], [67], [68]. In line with our result, genes involved in cell cycle control, DNA replication, molecular transport and cytoskeleton structure were highly expressed in mice pre-B cells [46].

The pathway analysis suggest opposite respond in antigen processing and presentation pathway (KEGG:hsa04612 in Table S4) in Ramos cells compared to other cells. The first actors in the antigen processing pathway, namely proteasome activator 28 (PA28)-αβ and a core particle of 20S proteasome α3, were negligibly expressed in Ramos cells whereas the downstream components, such as metalloprotease component of the 26S proteasome, ER and secretory granule proteins (albumin, ERp60, GRP-78, GRP-94, GRP-170, Hsp60 and HSP90AB1), were highly expressed (see Figure 5). In the other cell types, the expression of these proteins is reversed suggesting silencing of the antigen presentation pathway. Interestingly, PA28 and DJ-1 were co-expressed (cluster 17 Fig. 3) as shown previously [15], [69], but their possible interaction is not known. In conclusion, many of the proteins involved act close together in different pathways (see Table S4) indicating a signalling network, rather than separate pathways.

### Early Events Upon Anti-IgM Stimulation: Regulation of Cell Membrane-Cytoskeleton

BCR comprises the membrane-bound Ig in association with the accessory signal transduction components Ig-αβ [4]. Upon anti-IgM stimulation, the components of BCR complex were down-regulated (IgL λ SSP 168 in Figure 4) [13], [14] followed by the regulation of four cytoskeleton related proteins; stathmin, plastin-2, cofilin-1 and pre-mRNA-processing factor 40 homolog A (HIP-10) (clusters 4R and 11R in Figure 4, GO:7101 and GO:51261 in Table 1 and GO:8092 in Table 2). The pI changes in the actin binding proteins; stathmin, plastin-2 and cofilin-1, interpret regulation via PTM. In the early stage 1–4 h, plastin-2 (SSP 42, 43 and 45 in Figure 2 and Figure 4), stathmin (SSP 219 and 223 in Figure 2 and Figure 4) and HIP-10 were regulated followed by the down-regulation of cofilin-1 at 4–16 h (see Figure 4). Cofilin-1 depolymerizes actin filaments thereby promoting actin turnover. In line with our results, differential expression of cytoskeleton associated proteins was recently shown in anti-IgM stimulated chronic lymphocytic leukaemia (CLL) cells [70]. Rearrangements in the cytoskeleton play a pivotal role in B cell activation (reviewed in [71]). Yet, we do not fully understand how the actin-binding proteins link co-stimulation in the membrane to rearrangements in the underlying cytoskeleton. In human T cells, plastin-2 and stathmin are phosphorylated and cofilin-1 is dephosphorylated in response to co-stimulation through T cell receptor/CD3 and CD2 or CD28, respectively [72], [73], [74], [75].

Downstream from the events related to membrane-cytoskeleton, Ramos B cells progress to gene expression with few proteomic changes at 9–16 h: Phosphoglycerate mutase (SSP 184) was down-regulated at 4–12 h followed by up- and down-regulation of 60 S acidic ribosomal protein P0 at 9–16 h, as shown previously [15], [38]. Since the expression of these proteins is isoforms specific, it suggests regulation of translation via PTM. Further, elongation factor 1-β (SSP 171) was down-regulated at 16 h.

### Proteins from Different Signalling Pathways Act Together at 24 h

After the relatively quiescent time period around 16 h, a variety of downstream events occurred at 24 h (see Figure 4). Proteins in Ras, FAS and cytoskeleton organization pathways were regulated among others. Many of these proteins are co-expressed also at 72 h (clusters 3R and 6R in Figure 4) reflecting the cell division cycle in Ramos cells. The regulation of myosin regulatory light chain 12A, actin, actin-related protein 2/3, PAK-2, ezrin and Rho-GDI2 indicate cytoskeleton remodelling (KEGG:hsa4810 in Table S4, clusters 3R, 5R and 6R in Figure 4). The up-regulation of G3BP-1, Rho-GDI2 and 14-3-3 β/α suggests activation of Ras pathway (GO:7265) at 24 h. The co-expression of PAK-2, Rho-GDI2 and lamin-B1 indicate FAS (CD95) signalling (BioCarta:fasPathway in Table S4), whereas the co-expression of 14-3-3 protein β/α, lamin-B1, PAK-2 and PA28γ indicate apoptosis (Reactome:578) at 24 h. The apoptosis in Ramos cells reaches a maximum at 24 h [76], but the mechanism is likely FAS independent [77], [78], [79]. Our results suggest that BCR activation is a dynamic network, in which proteins from different signalling pathways act closely together. Interestingly, proteins co-expressed at 24 h, namely ezrin, 14-3-3 τ and heterogenous nuclear ribonucleoprotein K (hnRNP K), act also in the very early phase upon anti-IgM stimulation [37], [80], [81], [82]. Changes in the phosphorylation status of these proteins are the major regulators of activation. For example, ezrin dephosphorylates in few minutes upon anti-IgM stimulation and has a conformational change, which detaches it temporarily from actin and from lipid rafts [37]. Syk is activated via Tyr-phosphorylation, but recent studies indicate that Syk is also Ser-phosphorylated at multiple sites and binds 14-3-3 proteins [80], [81]. Our time scale analysis suggests that those proteins known to be important in the very early stages upon BCR stimulation are re-regulated in 24 hours.

The equipment of Ramos B cells with larger quantities of ribosomal subunits and RNA binding proteins (GO:30529 in Table 3 and GO:3723 Table 2) indicate active translation at 24 and 72 h (Reactome:71 for gene expression). Trp-tRNA ligase (TrpRS) and Tyr-tRNA ligase (TyrRS) were up-regulated at 24 h, whereas eIF-2-α was continuously up-regulated 24–72 h (Figure 4). EIF-2-α forms a complex with GTP and initiator tRNA at the early steps of protein synthesis. Interestingly, TrpRS had identical expression profile with PA28γ. The role of PA28γ in B-cells is not clear, although its up-regulation is shown previously in stimulated B cells indicating its importance in B cell activation [38]. In line with our results, the regulation of hnRNP proteins (ribonucleoprotein complex GO:30529 in Table 3) is shown in a study of stimulated CLL cells [70].

In general, our results are in accordance with transcriptomic results, since mRNA expression of genes involved in signal transduction and transcription was up-regulated after 24 h [12], [13], [14]. However, a more detailed analysis revealed differences between the expressions of corresponding genes/proteins. For example, 14-3-3 proteins τ, β/α and ε (GO:35308 in Table 1, GO:50815 and GO:42826 in Table 2) were co-regulated at proteomic level, but at transcriptomic level their mRNA profiles differ [14].

### A Turning Point after 72 h with Decreased Energy Requirements

As seen in Figure 4, 48 h was a relatively quiescent time point with a continuum of down-regulation events that begun at 24 h. Whereas 72 h was a turning point with two kinds of cellular events. Firstly, some of the events that occurred at 24 h reoccur at 72 h, like mRNA translation (see clusters 3R and 6R in Figure 3 and ribonucleoprotein complex GO:30529 in Table 3), reflecting the cell cycle phase in Ramos B cells. Secondly, proteins involved in hemopoiesis (GO:30097 in Table 1) were down-regulated and energy production begun to decrease. For example, the down-regulation of enzymes involved in tricarboxylic acid cycle (Reactome:1046 in Table S4) and metabolism of oxaloacetate and carbohydrate (GO:6107, GO:44262 and GO:6096 in Table 1) indicate decreased metabolism after 72 h of stimulation until the end of the time course. The result suggests a dual respond at 72 h: One population of Ramos B cells entered the cell cycle whereas the other population with decreased metabolism was prevented of entering the cell cycle but committed to apoptosis. In line with our result, the ligation of IgM triggers B cells into proliferation and apoptosis [76], [83], [84], [85]. The proportion of Ramos B cells, which enter into apoptosis after crosslinking of IgM, depends on the phase of the cell cycle and the affinity of the anti-IgM [76], [85]. The down-regulation of genes related to metabolism, cell growth, cell division and DNA synthesis was seen also at transcriptomic level late in the time course [12], [13], [14].

### Conclusions

The goal of the proteomic analysis of B cells was to identify the major protein expression effects during the maturation pathway. Our study revealed co-expression and -regulation of nearly 190 proteins related to the cellular processes and signalling pathways in the B cell differentiation pathway from early pre-B to terminally differentiated plasma cell stage. Our data and analysis provide a new and deeper description of the B cell proteome during maturation and in response to BCR activation compared to previous results thereby extending and complementing our knowledge of B cell ontogeny. We showed the major changes in functions of co-expressed proteins between the maturation stages with greatest difference between early pre-B/pre-B and IM-B stages. In Ramos and U-266 cells, the different combinations of proteins in Ig secretory machine indicate partly overlapping, but distinctive systems for Ig processing and transport. Our time course analysis of Ramos cells upon anti-IgM stimulation exposed the early regulation of membrane-cytoskeleton –linked proteins and the activation of many downstream signalling cascades in 24 h, like FAS and cytoskeleton rearrangement. The results suggest that many proteins known to be important in the early steps upon BCR stimulation are re-regulated in 24 h. We showed the 72 h turning point, after which the metabolic processes were silenced indicating cell death in Ramos B cells.

Additional studies are required to determine the role of the new protein candidates, such as UBLCP1 in pre-B cells (697, Nalm-1 and Nalm-6) and VAT-1 and MAGE 4 in plasma cells (U-266). Also, PTMs in cytoskeleton linked proteins, like plastin-2, stathmin and cofilin-1 should be investigated further, to elucidate the BCR activation mechanism in detail.

In conclusion, our proteomic and transcriptomic results for anti-IgM stimulated Ramos cells are in accordance, since specific cellular events take place in the same time points. But there are also cases where the mRNA and protein levels differ. The differences in protein and mRNA expression are widely known and reported in many comparative studies [86], [87], [88], [89]. Since many cellular processes are regulated at the protein level, as noticed also in our previous 2D-GE study [15], it is important to study the expression dynamics directly at proteome level. This approach provides deeper insights into B cell ontogeny and may open new avenues for novel therapeutic strategies to treat PID patients.

## Supporting Information

Figure S1 **Unsupervised clustering and correlation analyses of B cell samples on the basis of proteome profiles.** Two-way hierarchical cluster analysis and correlation matrix of the B cell lines based on protein abundance profiles of 2063 protein spots in relative to internal standard. The degree of similarity in profiles is shown by colour (scales below figure). Anti-IgM stimulated Ramos cells clustered into three subgroups according to time (1–4, 9–24 and 48–120 h) and the nearby time points correlate strongly. Early pre-B (RS4;11, 380 and REH) and pre-B cells (697, Nalm-1 and Nalm-6) have positive correlation. Early pre-B/pre-B and Ramos cells at late time points (48–96 h) have weak negative correlation. U-266 plasma cell is distinctive from the other cell types.
(PDF)

Figure S2 **Cluster dendrogram of B cell samples on the basis of proteome profile.** The dendrogram shows the distinct pattern of IM-B Ramos cell samples (right branch) compared to early pre-B (380, REH and RS4;11), pre-B (697, Nalm-1, Nalm-6) and U-266 plasma cell samples (left branch). The stimulated and corresponding control Ramos B samples are clustered in twos indicating minor anti-IgM induced proteome-wide changes, except at 24 h (clustered singly) and 96–120 h (clustered two by two).
(PDF)

Table S1 **B cell lines.** List of B cell lines with respective origin, differentiation stage and Ig chain expression.
(PDF)

Table S2 **Proteins in B cell proteome database.** List of identified proteins with respective UniProt accession numbers, experimental pI and Mr values, number of peptide matches, intensity and sequence coverage, as well as Mascot scores to show the reliability of the identifications (p<0.05).
(PDF)

Table S3 **Peptides identified from different proteins by fragment ion analysis.** List of identified peptides with Mascot scores to show the reliability of the identifications (p<0.05).
(PDF)

Table S4 **Signalling pathways and identified proteins involved.** List of Reactome, KEGG and BioCarta pathways and proteins involved with corresponding p-values.
(PDF)
