# Supplementary figures and images for: Proteomic Changes during B Cell Maturation: 2D-DIGE Approach
Source: PLoS One. 2013 Oct 29;8(10):e77894. doi: 10.1371/journal.pone.0077894 (PMC3812168; doi:10.1371/journal.pone.0077894)

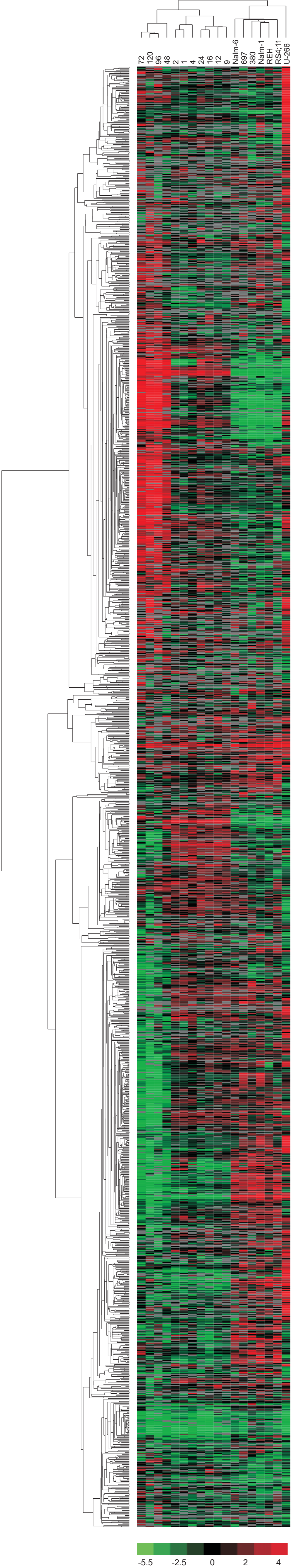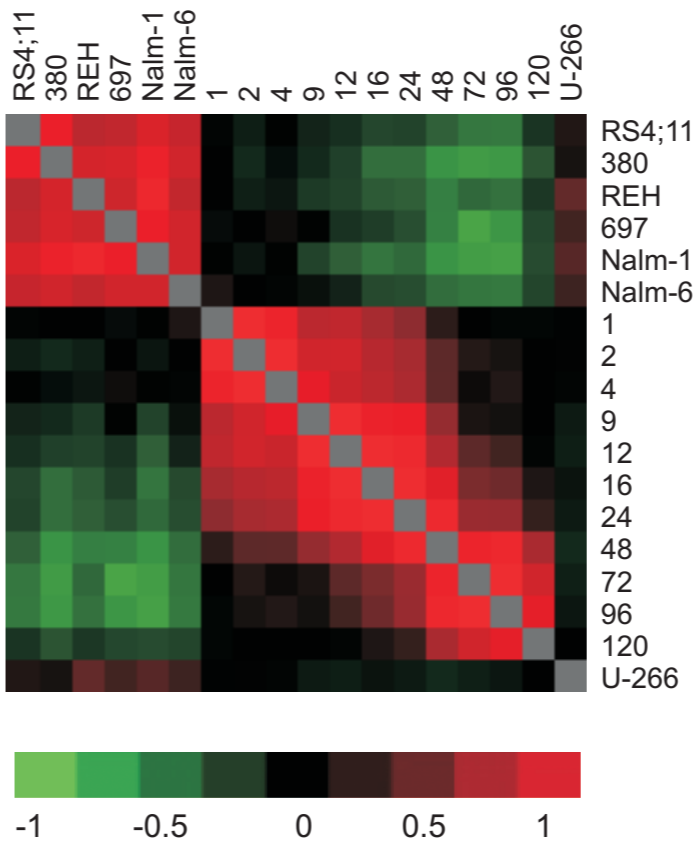

Supplement: Figure S1 — Unsupervised clustering and correlation analyses of B cell samples on the basis of proteome profiles. Two-way hierarchical cluster analysis and correlation matrix of the B cell lines based on protein abundance profiles of 2063 protein spots in relative to internal standard. The degree of similarity in profiles is shown by colour (scales below figure). Anti-IgM stimulated Ramos cells clustered into three subgroups according to time (1–4, 9–24 and 48–120 h) and the nearby time points correlate strongly. Early pre-B (RS4;11, 380 and REH) and pre-B cells (697, Nalm-1 and Nalm-6) have positive correlation. Early pre-B/pre-B and Ramos cells at late time points (48–96 h) have weak negative correlation. U-266 plasma cell is distinctive from the other cell types. (PDF) [file pone.0077894.s001.pdf]

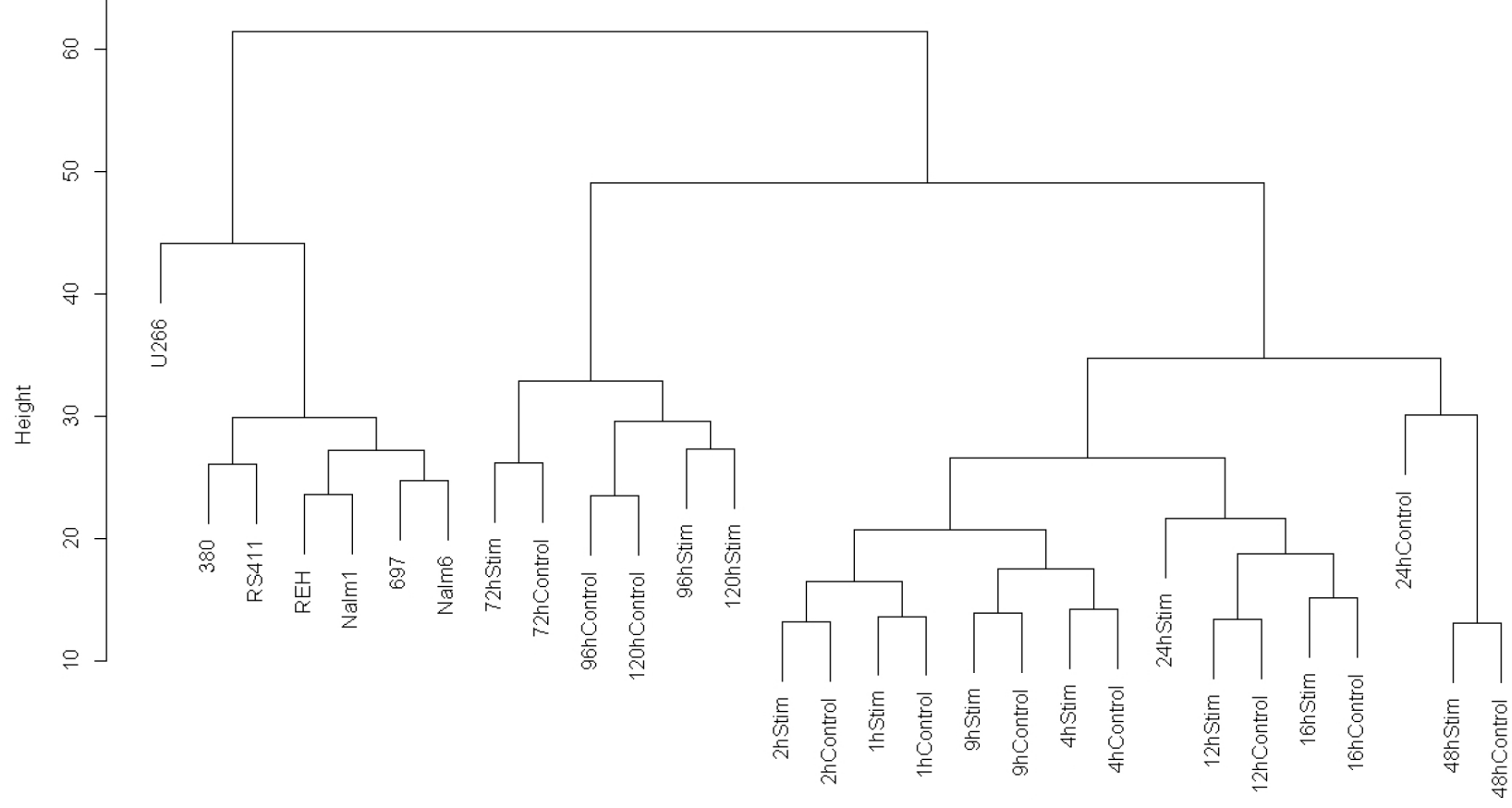

Supplement: Figure S2 — Cluster dendrogram of B cell samples on the basis of proteome profile. The dendrogram shows the distinct pattern of IM-B Ramos cell samples (right branch) compared to early pre-B (380, REH and RS4;11), pre-B (697, Nalm-1, Nalm-6) and U-266 plasma cell samples (left branch). The stimulated and corresponding control Ramos B samples are clustered in twos indicating minor anti-IgM induced proteome-wide changes, except at 24 h (clustered singly) and 96–120 h (clustered two by two). (PDF) [file pone.0077894.s002.pdf]
