# Supplementary material for: Proteomic Changes during B Cell Maturation: 2D-DIGE Approach
Source: PLoS One. 2013 Oct 29;8(10):e77894. doi: 10.1371/journal.pone.0077894 (PMC3812168; doi:10.1371/journal.pone.0077894)
Supplement: Table S1 — B cell lines. List of B cell lines with respective origin, differentiation stage and Ig chain expression. (PDF) [file pone.0077894.s003.pdf]

Supplementary Table S1. B cell lines. List of B cell lines with respective origin, differentiation stage and Ig chain expression.

B cell line	Stage	DSMZ no[a)]	Ig	Patient	Year	Source	Disease	Reference
**RS4;11**	early pre-B	ACC-508	IgM–	32 F	1985	BM	ALL L2	[1]
**380**	early pre-B	ACC-39	IgM–	15 M	1983	PB	ALL FAB L2	[2]
**REH**	early pre-B	ACC-22	IgM–	15 F	1973	PB	ALL	[3]
**697**	pre-B	ACC-42	IgM cy+	12 M	1979	BM	null-ALL	[4]
**Nalm-1**	pre-B	ACC-131	IgM cy+	3 F	1975	PB	CML	[5]
**Nalm-6**	pre-B	ACC-128	IgM cy+	19 M	1976	PB	ALL	[6]
**Ramos**	immature B	ACC-603	IgM sm/cy+	3 M	1972	PB	BL	[7]
**U-266**	plasma cell	ACC-9	IgM–, IgE s+	53 M	1968	PB	MM	[8]

a) Accession number in Leibniz-Institute DSMZ-German Collection of Microorganisms and Cell Cultures (https://www.dsmz.de).

Abbrevations: ALL acute lymphoblastic leukemia, BL Burkitt lymphoma, BM bone marrow, CML chronic myeloid leukemia, cy cytosolic, F femail, PB peripheral blood, s secreted, sm cell surface membrane, M male, MM multiple myeloma

[1] Stong RC, Korsmeyer SJ, Parkin JL, Arthur DC, Kersey JH (1985) Human acute leukemia cell line with the t(4;11) chromosomal rearrangement exhibits B lineage and monocytic characteristics. Blood 65: 21-31.

[2] Pegoraro L, Palumbo A, Erikson J, Falda M, Giovanazzo B, et al. (1984) A 14;18 and an 8;14 chromosome translocation in a cell line derived from an acute B-cell leukemia. Proc Natl Acad Sci U S A 81: 7166-7170.

Supplementary Table S1. B cell lines. List of B cell lines with respective origin, differentiation stage and Ig chain expression.

[3] Rosenfeld C, Goutner A, Choquet C, Venuat AM, Kayibanda B, et al. (1977) Phenotypic characterisation of a unique non-T, non-B acute lymphoblastic leukaemia cell line. Nature 267: 841-843.

[4] Findley HW Jr, Cooper MD, Kim TH, Alvarado C, Ragab AH (1982) Two new acute lymphoblastic leukemia cell lines with early B-cell phenotype. Blood 60: 1305-1309.

[5] Minowada J, Koshiba H, Janossy G, Greaves MF, Bollum FJ (1979) A Philadelphia chromosome positive human leukaemia cell line (NALM-1) with pre-B characteristics. Leuk Res 3: 261-266.

[6] Hurwitz R, Hozier J, LeBien T, Minowada J, Gajil-Peczalska K, et al. (1979) Characterization of a leukemic cell line of the pre-B phenotype. Int J Cancer 23: 174-180.

[7] Klein G, Giovanella B, Westman A, Stehlin JS, Mumford D (1970) An EBV-genome-negative cell line established from an American Burkitt lymphoma; receptor characteristics. EBV infectibility and permanent conversion into EBV-positive sublines by in vitro infection. Intervirology 5: 319-334.

[8] Nilsson K, Bennich H, Johansson SG and Ponten J (1970) Established immunoglobulin producing myeloma (IgE) and lymphoblastoid (IgG) cell lines from an IgE myeloma patient. Clin Exp Immunol 7: 477-489.
