# Supplementary material for: Proteomic Changes during B Cell Maturation: 2D-DIGE Approach
Source: PLoS One. 2013 Oct 29;8(10):e77894. doi: 10.1371/journal.pone.0077894 (PMC3812168; doi:10.1371/journal.pone.0077894)
Supplement: Table S2 — Proteins in B cell proteome database. List of identified proteins with respective UniProt accession numbers, experimental pI and Mr values, number of peptide matches, intensity and sequence coverage, as well as Mascot scores to show the reliability of the identifications (p<0.05). (PDF) [file pone.0077894.s004.pdf]

Supplementary Table S2. **Proteins in B cell proteome database**. Proteins are identified either by PMF or by combining MS and MS/MS spectral data (Supplementary Table S3) for database searches. Identifications were confirmed by operator inspection.

Protein name (abbreviation)	SSP[a)]	Accession[b)]	Identification[c)]	Mascot score[d)]	Intensity coverage (%)	Sequence coverage (%)	Peptide matches	Mr	pI	Status[e)]	2D-GE DB SSP[f)]
14-3-3 protein-β/α	185	P31946	PMF	75	71		6			1R	null
14-3-3 protein-ε	172	P62258	PMF comb.	69	28	36	9	31	4	1R	71
14-3-3 protein-τ	180	P27348	PMF	114	74	35	10			17, 7R	null
26S proteasome non-ATPase regulatory subunit 14	155	O00487	PMF	74	45	30	6	35	6.3	2	68
40S ribosomal protein SA	130	P08865	PMF	147	92	41	10			1R	null
60S acidic ribosomal protein P0 (RPLP0)	146	P05388	PMF	156	54	33	13	36	5.5	11R	64
	148	P05388	PMF comb.	158	64	10		36	6.1	1, Ramos	65
	150	P05388	PMF	147	92	41	10		6.1	1, Ramos	65
60 kDa heat shock protein, mitochondrial (Hsp60)	54	P10809	PMF	94	31	27	14	64	5	3	25
	55	P10809	PMF		83	43	17	64	5.1	3	26
Acidic leucine-rich nuclear phosphoprotein 32 family member A	179	P39687	PMF	99	48	38	7	29	3.5	bkg	80
β-Actin	112	P60709	PMF	143	52	52	18	44	5	3R	54
	113	P60709	PMF			50				bkg	55
	114, 115	P60709	PMF	201	92	43	15			15, 3R	null
	121	P60709	PMF			22	6	42	5.7	5R	56
	122	P60709	PMF	174	89	41	13	42	5.9	5R	57
	126	P60709	PMF			50	15	43	5.1	11	55
	127	P60709	PMF			50	15	43	5.1	3R	55
	162	P60709	PMF	78	57	24	7	33	4.4	B, 7R	72
	173	P60709	PMF	98	18	79	9	30	5.1	B, 6R	74
	186	P60709	MS/MS	80				28	5.3	bkg	83
γ-Actin	198	P63261	PMF	119	92	38	7	26	5.5	6R	95

Supplementary Table S2. **Proteins in B cell proteome database**. Proteins are identified either by PMF or by combining MS and MS/MS spectral data (Supplementary Table S3) for database searches. Identifications were confirmed by operator inspection.

Actin-related protein 2/3 (Arp 2/3) complex subunit 5-like protein	221	Q9BPX5	PMF comb.	114	88	21	3			3, 1R	null
Actin-related protein 3	94	P61158	PMF	254	84	55	22	48	5.6	bkg	49
ACTN3 protein	182	Q4VAM3	PMF	71	39	16	8	30		bkg	null
Adenosylhomocysteinase	117	P23526	PMF	126	83	23	11	44	6.3	16	58
Annexin A5 (AnxA5)	158	P08758	PMF	141	66	33	11			12	null
AnxA6	38	P08133	PMF	340	91	48				12, Ramos	null
Aspartate aminotransferase, mitochondrial (mAspAT)	129	P00505	PMF	194	87	16				4, 10R	null
Aspartyl-tRNA synthetase, cytoplasmic	78	P14868	PMF	159	91	25	11	54	6.7	bkg	43
ATP-dependent RNA helicase DDX39	72	O00148	PMF	107	73	22	10	55	5.4	bkg	39
ATP synthase subunit β, mitochondrial	93	P06576	PMF			47	18	50	4.7	2	44
Bifunctional purine biosynthesis protein PURH	47	P31939	PMF	129	65	29	16	68	6.7	16	23
Biliverdin reductase A	144	P53004	PMF comb.	70	16	18	4	33	6.5	B	null
Bruton tyrosine kinase (Btk)	138	Q06187	PMF			23	10	39	6.9	16	66
Chloride intracellular channel protein 1	169	O00299	PMF	224	55	54	14			B	null
Chromobox protein homolog 2	80	Q14781	PMF	60	22	15	6			3	null
Citrate synthase, mitochondrial	118	O75390	PMF	70			7			10R	60
Cofilin-1	218	P23528	PMF comb.	63	56	39	5	19	6.6	bkg	null
	222	P23528	MS/MS							14, 10R	null
Coronin-1A	58	P31146	PMF				9			bkg	33
	59	P31146	PMF	149	46	36	15	60	6.6	15	34
	88	P31146	PMF	81	43	28	9	60		bkg	null
Cytochrome b-c1 complex subunit 1, mitochondrial	101	P31930	PMF	116	71	28		48	5.4	2	48
Cytoplasmic aconitate hydratase	15	P21399	PMF	93	97	10	8	99	6.2	14	null
Cytosol aminopeptidase	79	P28838	PMF	239	85	44	19	57	9	10	null
Cytosolic non-specific dipeptidase	84	Q96KP4	PMF	266	83	47	20			10	null
D-3-phosphoglycerate dehydrogenase	73	O43175	PMF	164	74	28	14	57	6.3	15	null
δ(3,5)-δ(2,4)-Dienoyl-CoA isomerase,	164	Q13011	PMF	99	79	26	7			4	null

Supplementary Table S2. **Proteins in B cell proteome database**. Proteins are identified either by PMF or by combining MS and MS/MS spectral data (Supplementary Table S3) for database searches. Identifications were confirmed by operator inspection.

mitochondrial											
	165	Q13011	PMF comb.	62			3			14	null
dUTPase, mitochondrial	215	P33316-2	PMF	81	51	46	6	20	5.7	15	103
Dihydrolipoyl dehydrogenase, mitochondrial	66	P09622	PMF comb.	126	45	15	6	56	6.9	2	36
DJ-1	210	Q99497	PMF comb.	54	36	31	4	23	6.3	17	102
DNA mismatch repair protein Msh2	4	P43246	PMF	64	80	8	7			14	null
DNA replication licensing factor MCM7	6	P33993	PMF			12	6	115	6.5	16	6
	19	P33993	PMF	229	89	29	20		6.5	17	null
Elongation factor 1-β (EF-1-β)	171	P24534	PMF	82	75	30	6			Ramos	null
EF-1-δ	154	P29692	PMF comb.	120	49	46	10			7	null
EF-2	7	P13639	PMF	112	63	18	13			15	null
	8	P13639	Match					111	6.8	15	7
	9	P13639	Match					111	6.8	15	8
	10	P13639	PMF			13	8	111	6.9	15	9
	11	P13639	Match					111	6.9	15	10
Endoplasmin (GRP-94)	12	P14625	PMF	285	84	41	37	113	4.3	8	2
α-Enolase	95	P06733	PMF	88	37	22	9			15	null
	96	P06733	PMF	159	52	49	19			B	null
	103	P06733	PMF	150	49	47		49	6.8	B, 9R	51
	104, 105, 110	P06733	PMF			49	16	48	6.9	15	52
α-Enolase	106	P06733	PMF	244	97	49	17			15, 4R	null
γ-Enolase	107	P09104	PMF	263	88	53	20			12	null
Enoyl-CoA hydratase, mitochondrial	190	P30084	PMF	79	37	28	6			bkg	null
ER resident protein 29 (ERp29)	183	P30040	PMF	92	74	29	7			14	null
Eukaryotic translation initiation factor 2 subunit 1 (eIF-2-α)	142	P05198	PMF comb.	68	25	14	4			2, 3R	null
eIF-5A-1	224	P63241	PMF comb.	135	51	49	5			18	null
Ezrin	21	P15311	PMF	143	63	29	17			15, 7R	null

Supplementary Table S2. **Proteins in B cell proteome database**. Proteins are identified either by PMF or by combining MS and MS/MS spectral data (Supplementary Table S3) for database searches. Identifications were confirmed by operator inspection.

F-actin capping protein subunit β	170	P47756	PMF	103	46	38	10	31	5.5	bkg	76
Fatty acid-binding protein, epidermal	228	Q01469	PMF	82	8	61	6			B	null
Fatty acid synthase	3	P49327	PMF	204	91	12	24			6R	null
Fructose-bisphosphate aldolase A	134	P04075	PMF	142	56	40	12			16, 9R	null
Fructose-bisphosphate aldolase C	132	P09972	PMF	165	70	49	12	40	6.9	bkg	62
Fumarate hydratase, mitochondrial	111	P07954	PMF	100	100	21	6	46	7.1	bkg	53
Galectin-1	230	P09382	PMF	141	43	63	8			12	null
78 kDa glucose-regulated protein (GRP-78)	25	P11021	PMF	372	87	54	30			8	null
	26	P11021	PMF	304	86	45	27			8	null
	27	P11021	PMF	206	86	36	18	72	4.9	8	null
Glutathione S-transferase ο-1	178	P78417	PMF	116	88	28	8			B	null
Glutathione S-transferase P	207	P09211	PMF comb.	85	52	49	7	25	5.1	bkg	93
	208	P09211	PMF	121	32	62	11	24	5.4	B	94
Glycyl-tRNA synthetase	23	P41250	PMF			16	8	83	6.2	B	12
Growth factor receptor-bound protein 2	200	P62993	PMF	129	83	38	9	26	6	bkg	97
GTP-binding nuclear protein Ran	211	P62826	PMF	95	52	45	7	24	7	bkg	100
Heat shock cognate 71 kDa protein	32	P11142-2	PMF			18	10	76	5.2	bkg	13
HSP 75, mitochondrial	29	Q12931	PMF	207	76	38	23	79	6.7	B	16
HSP 90-α	20	P07900	PMF comb.	134	62	32	13	95	4.7	bkg	3
HSP 90-β	20	P08238	PMF comb.	321	77	36	13	95	4.7	bkg	3
Heat-shock protein β-1 (HspB1)	197	P04792	PMF	168	96	54	10	26	6.1	18	88
	199	P04792	PMF	80	100	23	4	26	5.6	8R	96
Heterogeneous nuclear ribonucleoprotein (hnRNP) A2/B1	156	P22626	Match					34	9.1	bkg	70
hnRNP C1/C2	131	P07910	PMF comb.	128	88	33	5	19	4.7	1, 3R	null
	141	P07910	PMF comb.	68			2			2, 3R	null
	143	P07910	PMF comb.	92	57	21	4	19	4.7	6R	null
hnRNP H	85	P31943	PMF	155	66	46	15	52	5.9	bkg	46
	86	P31943	PMF	96	76	29	8	52	6	bkg	null
hnRNP K	53	P61978	PMF	140	79	33	14	65	5	B	24

Supplementary Table S2. **Proteins in B cell proteome database**. Proteins are identified either by PMF or by combining MS and MS/MS spectral data (Supplementary Table S3) for database searches. Identifications were confirmed by operator inspection.

	71	P61978	PMF comb.	131	42	24	8	48	5.2	B, 2R	null
hnRNP Q	116	O60506	PMF	84	79	10	7			6R	null
HLA class II histocompatibility antigen, DR β 3 chain	204, 205	Q860I9	PMF	64	11	73	5	25	7	bkg	99
HSPA9	33	Q8N1C8	PMF			17	9	76	5.4	16	14
HSP90AB1	153	Q6PK50	PMF comb.	87		8	3	40	4.9	6, 6R	null
Hydroxymethylglutaryl-CoA (HMG-CoA) synthase, cytoplasmic	69	Q01581	PMF	109	41	30	11			B, 1R	null
	70	Q01581	PMF	152	66	34	14		5.1	B, 1R	null
Hypoxia up-regulated protein 1 (GRP-170)	1	Q9Y4L1	PMF	109	40	28	22	150	5	14	1
	2	Q9Y4L1	PMF	184	89	21	20	150		14	null
Ig λ	168	P04211, P0CG04	PMF	89			6	30	4.9	6, 8R	
Ig λ, VLJ region	175	P04209	PMF comb.	74	12	36	5			6	null
Ig μ, C region	30	P01871	PMF	141	76	28	12	80		6	null
	31	P01871	PMF	168	77	24	14	80		6	null
Importin subunit α-2	61	P52292	PMF	81	42	22	9			bkg	null
Inosine-5´-monophosphate dehydrogenase 2 (IMPD 2)	65	P12268	PMF	68	21	29	10	59	6.8	11	35
Isocitrate dehydrogenase [NAD] subunit α, mitochondrial	136	P50213	PMF	138	84	31	11	38	5.9	15	61
Lamin-B1	40	P20700	PMF	68	16	30	11	75	4.9	18	17
	41	P20700	PMF	120	47	29	13	72	5	19, 1R	18
Leukocyte elastase inhibitor	123	P30740	PMF	111	79	27	9	43	5.9	B	null
L-lactate dehydrogenase B chain (LDH-B)	147, 149	P07195	PMF comb.	46	21	19	6	35	5.7	16, 9R	67
LOC728395 protein	135	A4FUW6	PMF	119	85	31	8			B	null
Malate dehydrogenase, cytoplasmic (Mdh1)	151	P40925	PMF	83	85	22	6	36	6.8	16	69

Supplementary Table S2. **Proteins in B cell proteome database**. Proteins are identified either by PMF or by combining MS and MS/MS spectral data (Supplementary Table S3) for database searches. Identifications were confirmed by operator inspection.

Mdh2	152	Q6FHZ0	PMF	168	66	51	16	36	9.8	B, 10R	null
Melanoma-associated antigen 4 (MAGE 4)	119	P43358	PMF	134	93	31	9			14	null
	120	P43358	PMF	117	91	29	8			14	null
Mitochondrial aldehyde dehydrogenase 2	75	Q53FB6	PMF	209	91	40	15			6	null
Moesin	24	P26038	PMF	149	73	34	18			11	null
Myosin regulatory light chain 12A	216	P19105	PMF comb.	46						6R	null
Neutral α-glucosidase AB	5	Q14697-1	PMF	105	82	11	10		5.9	9	null
	14	Q14697	PMF	297	97	40	27	110	5.8	9	5
Non-specific lipid-transfer protein (NSL-TP), isoform CSP2	231	P22307-2	PMF	131	76	19	8			6	null
NSL-TP	232	P22307-2	PMF	131	76	19	6			6	null
Nucleophosmin	140, 145	P06748	PMF	65	62	31	6	37	4.2	bkg	63
Nucleoside diphosphate kinase A (NDK A)	217	P15531	PMF		83	57	11	19	5.9	16, 9R	104
Peptidyl-prolyl cis-trans isomerase A	225	P62937	PMF	120	68	59	11	16	7.1	15	108
	226	P62937	Match					16	7.6	15	109
Peroxiredoxin (Prdx-1)	212	Q06830	PMF	144	59	71	10	23	7.4	19	101
	213	Q06830	PMF	122	72	44	8		8.8	11	null
Prdx-2	214	Q06830	PMF	100	82	40	6			bkg	null
Prdx-4	189	Q13162	PMF	89	40	51	8	27	5.6	5	84
Plastin-2	42	P13796	PMF	144	93	21	12		4.9	B, 11R	null
	43	P13796	PMF	210	80	34	19	69	5	B, 11R	20
	44	P13796	PMF	69	50	12	9	71	5.1	B, 4R	21
	45	P13796	PMF	89	77	21	12		5.2	B, 4R	null
Phosphoglycerate mutase 1	184	P18669	PMF	107	52	44	8	29	6.6	14, 11R	86
	187	P18669	PMF	193	97	51	12	28	6.9	17	87
	191	P18669	PMF	254	55	70	19	28	6.1	bkg	85
Phosphoserine aminotransferase	133	Q9Y617	PMF	70	34	20	7	41	8.7	B	null
Pre-mRNA-processing factor 40 homolog A (HIP-10)	28	O75400	PMF	77	77	20	6	90		B, 4R	null

Supplementary Table S2. **Proteins in B cell proteome database**. Proteins are identified either by PMF or by combining MS and MS/MS spectral data (Supplementary Table S3) for database searches. Identifications were confirmed by operator inspection.

Profilin-1	229	P07737	PMF comb.	95	27	37	4			13	null
Prohibitin	176	P35232	PMF	229	96	34	13	29	5.1	2	82
Proliferating cell nuclear antigen (PCNA)	157	P12004	PMF	90	62	30				11	null
Proteasome activator 28 subunit α (PA28α)	177	Q06323	PMF	107	46	37	9	30	5.7	17	77
PA28β	174	Q9UL46	PMF	141	64	46	12	31	5.2	17	75
PA28γ	163	P61289	PMF	91	54	25	7			3R	null
Proteasome subunit α type 2	203	P25787	Match					25	6.9	bkg	98
Proteasome subunit α type 3	181	P25788	PMF	116	60	38	9	29	5	15, 8R	81
Proteasome subunit α type 7	188	O14818	PMF	86	73	25	6	28	9.3	9	null
Protein disulfide-isomerase (PDI)	60	P07237	PMF	264	72	42	22			8	null
Protein disulfide-isomerase A3 (ERp60)	62	P30101	PMF	176	65	45	18	58	5.7	14	31
	63	P30101	PMF			40	16	57	5.8	14	32
	64	P30101	PMF	181	69	37	18		5.9	6	null
Protein disulfide-isomerase A6 (ERp5)	97	Q15084	PMF	201	86	45	15			9	null
Rab GDP dissociation Inhibitor β	102	P50395	PMF	198	94	34	18	48	6.4	16	50
Ras GTPase-activating protein-binding protein 1 (G3BP-1)	49	Q13283	PMF	123	35	33	10	52	5.3	B, 3R	null
Rho GDP-dissociation inhibitor 1 (Rho GDI 1)	202	P52565	Match					26	4.6	bkg	91
Rho GDI 2	206	P52566	PMF comb.	83	6	25	3	25	4.9	19, 1R	92
RuvB-like 1	77	Q9Y265	PMF	152	62	35	12	54	6.4	2	41
	87	Q9Y265	PMF comb.	60	25	25	8	53	6.5	3	42
RuvB-like 2	89	Q9Y230	PMF	94	63	20	9		5.4	2, 5R	null
	90	Q9Y230	PMF			29	12	50	5.5	2	45
Septin-2	124	Q15019	PMF	130	80	34	12	43	6.5	15	59
Serum albumin	34	P02768	PMF comb.	30			3			B, 7R	null
	35	P02768	PMF comb.	87			5			3, 7R	null
	36	P02768	PMF	101	84	16	9		5.8	3, 7R	null
Ser/Thr-protein kinase PAK 2 (PAK-2)	57	Q13177	PMF	119	59	35	11			15, 2R	null
SET	125	Q01105	PMF	90	82	25				10, 9R	null

Supplementary Table S2. **Proteins in B cell proteome database**. Proteins are identified either by PMF or by combining MS and MS/MS spectral data (Supplementary Table S3) for database searches. Identifications were confirmed by operator inspection.

SET nuclear oncogene	139	Q5VXV2	PMF	138	92	26				14, 9R	null
S-formylglutathione hydrolase	166	P10768	PMF	140	85	44	10			bkg	null
Small nuclear ribonucleoprotein F	233	P62306	MS/MS	34			2			bkg	null
Sortin nexin-6	91	Q9UNH7	PMF	90	61	28	9			bkg	null
Succinate dehydrogenase [ubiquinone] flavoprotein subunit mitochondrial (Fp)	39	P31040	PMF	112	92	17	8	72	6.6	15	19
Superoxide dismutase [Cu-Zn] (Sod1)	220	P00441	PMF	54				19	5.7	14, 9R	106
Spermidine synthase	159	P19623	PMF	89	6	19	6	34	5.1	bkg	73
Spliceosome RNA helicase DDX39B	82	Q13838	PMF comb.	102	66	24	9	54	5.4	18	38
	83	Q13838	PMF	82	77	19	8	49	5.5	11, 3R	null
Stathmin	219	P16949	PMF	70	48	38	7		5.3	14, 11R	null
	223	P16949	MS/MS					18	5.6	16, 4R	107
Synaptic vesicle membrane protein VAT-1 homolog	109	Q99536	PMF	88	82	25	7			10	null
T-complex protein 1α	50	P17987	PMF			31	14	64	6	bkg	28
T-complex protein 1ε	48	P48643	PMF	124	60	38	19	64	5.4	bkg	27
T-complex protein 1γ	46	P49368	PMF	196	85	36	16	68	6.4	bkg	22
T-complex protein 1τ	56	P50990	PMF	190	85	37	17	62	5.4	bkg	30
T-complex protein 1ζ	51	P40227	PMF	188	85	29	13	65	6.7	bkg	29
Thioredoxin domain-containig protein 5 (ERp46)	98	Q8NBS9	PMF	194	87	27	13			9	null
Transaldolase	137	P37837	PMF comb.	72	30	14	4			15	null
Transitional ER ATPase	16	P55072	Match					100	5	bkg	4
Trifunctional purine biosynthetic protein adenosine-3 (PUR2)	92	P22102	PMF comb.	63	30		4			3R	null
Triosephosphate isomerase	193	P60174	Match					27	6.7	15	89
	194	P60174	PMF	326	92	88	21	27	6.9	15	90
Tropomyosin α-3 chain isoform 3	161	P06753	PMF comb.	121	35	28	7			11	null
Tropomyosin α-4 chain	160	P67936	PMF	91	33	31	10			11	null
Tryptophanyl-tRNA synthetase (TrpRS)	76	P23381	PMF			22	8	56	6.1	B	40
Tubulin α	81	P05209	PMF	138	48	48	15	50	5.3	bkg	37

Supplementary Table S2. **Proteins in B cell proteome database**. Proteins are identified either by PMF or by combining MS and MS/MS spectral data (Supplementary Table S3) for database searches. Identifications were confirmed by operator inspection.

Tumor protein D52	201	P55327	PMF	147	11	63	8	20	4.8	B	null
Tyrosyl-tRNA synthetase, cytoplasmic (TyrRS)	52	P54577	PMF	176	55	28	14			3R	null
TXNDC5 protein	99	Q86UY0	PMF	190	71	56	16			9	null
	100	Q86UY0	PMF	195	60	54				9	null
	108	Q86UY0	PMF	138	75	44	12			9	null
Ubiquitin carboxyl-terminal hydrolase isozyme L1	195	P09936	PMF	64	27	42	6			B	null
	196	P09936	PMF	168	51	70				10	null
Ubiquitin carboxyl-terminal hydrolase isozyme L3	192	P15374	PMF	144	88	37	9			bkg	null
Ubiquitin carboxyl-terminal hydrolase 5	13	P45974-2	PMF	97	38	19	12			bkg	null
Ubiquitin-like domain-containing CTD phosphatase 1	128	Q8WVY7	PMF	64	57	15	6			B	null
UPF0587 protein C1orf123	209	Q9NWV4	PMF							B	null
UPF0556 protein C19orf10	227	Q969H8	PMF	70	46	27	4	15	6.7	10	110
UV excision repair protein RAD23 homolog B (HR23B)	74	P54727	PMF	94	84	24	7			B	null
Vasohibin-2	22	Q86V25-3		54						bkg	null
Vimentin	67	P08670	PMF	236	91	41	18			14	null
	68	P08670	PMF	338	80	66	28			14	null
Voltage-dependent anion-selective channel protein 1 (VDAC-1)	167	P21796	PMF	149	54	52	11	32	9	3	79
X-ray repair cross-complementing protein 5 (Ku80)	17	P13010	PMF	117	74	18	11		5.7	8R	null
	18	P13010	PMF	180	65	34	17		5.9	17	null
X-ray repair cross-complementing protein 6 (Ku70)	37	P12956	PMF	88	56	17	9	75	6.7	bkg	15

a) Sample spot number (SSP) refers to those in Figures 2 and 4, and Tables 1-3, S3 and S4.

b) Accession number in UniProt database.

Supplementary Table S2. **Proteins in B cell proteome database**. Proteins are identified either by PMF or by combining MS and MS/MS spectral data (Supplementary Table S3) for database searches. Identifications were confirmed by operator inspection.

c) Proteins were identified by PMF, by combining PMF and fragment ion analysis (PMF comb.), by fragment ion analysis (MS/MS) or by matching in lymphocyte 2D-GE databases (Match).

d) Mascot score shows the reliability of the identifications ($p<0.05$) in the database searches. High score indicates a likely identification.

e) Status referring to cluster numbers in Figures 3 and 4, and Tables 1-3 and S4. Ramos and B indicate that the protein exists in the Ramos and B cell dataset (Figures 4 and 3, respectively), but outside numbered clusters. Background (bkg) indicates that the protein abundance did not change.

f) SSP referring to 2D-GE database http://structure.bmc.lu.se/BcellProteome/ (Salonen et al., (2006). Proteomics 6: 5152-5168). Null indicates a new identification that does not exist in the 2D-GE database.
