# Supplementary material for: Proteomic Changes during B Cell Maturation: 2D-DIGE Approach
Source: PLoS One. 2013 Oct 29;8(10):e77894. doi: 10.1371/journal.pone.0077894 (PMC3812168; doi:10.1371/journal.pone.0077894)
Supplement: Table S3 — Peptides identified from different proteins by fragment ion analysis. List of identified peptides with Mascot scores to show the reliability of the identifications (p<0.05). (PDF) [file pone.0077894.s005.pdf]

Supplementary Table S3. Peptides identified from different proteins by fragment ion analysis.

Protein name	SSP[a)]	Accession[b)]	Mascot score[c)]	Observed mass [MH]+	Peptide sequence	Modification
Arp 2/3	198	Q9BPX5	33	785.483	R.AFHAALR.N	None
	198	Q9BPX5	26	1126.732	K.ALAVGGLGSIIR.V	None
Biliverdin reductase A	144	P53004	41	832.498	K.FGVVVVGVGR.A	None
Cofilin-1	222	P23528	64	1337.63	YALDATYETK	None
δ(3,5)-δ(2,4)-Dienoyl-CoA isomerase, mitochondrial	165	Q13011	18	1542.777	K.EVDVGLAADVGTLQR.L	None
EF-1-δ	154	P29692	41	973.653	K.LVPVGYGIR.K	None
Eukaryotic translation initiation factor 2 subunit 1 (eIF-2-α)	142	P05198	36	1348.701	K.VVTDTDETELAR.Q	None
eIF-5A-1	224	P63241	98	1298.787	K.VHLVGIDIFTGK.K	None
hnRNP C1/C2	131	P07910	64	943.634	R.VPPPPPIAR.A	None
	141	P07910	68	943.593	R.VPPPPPIAR.A	None
	143	P07910	49	943.644	R.VPPPPPIAR.A	None
hnRNP K	71	P61978	55	1194.749	R.NLPLPPPPPPR.G	None
HSP90AB1	153	Q6PK50	40	1194.745	IDIIPNPQER	None
Profilin-1	229	P07737	15	1379.716	K.STGGAPTFNVTVTK.T	None
	229	P07737	22	1641.733	R.DSLLQDGEFSMDLR	Oxidation (M)
Serum albumin	34	P02768	36	927.532	K.YLYEIAR.R	None
	36	P02768	50	927.424	K.YLYEIAR.R	None
Small nuclear ribonucleoprotein F	233	P62306	34	1051.621	R.CNNVLYIR.G	None
Transaldolase	137	P37837	38	1119.658	K.FAADAVKLER.M	None
Tropomyosin α-3 chain isoform 3	161	P06753	59	1727.852	R.IQLVEEELDRAQER.L	None

a) Sample spot number (SSP) refers to those in Figures 2 and 4 and Tables 1-3, S2 and S4.

b) Accession number in Uniprot database.

c) Individual ion scores indicate identity or extensive homology ($p<0.05$).
