# Supplementary material for: Proteomic Changes during B Cell Maturation: 2D-DIGE Approach
Source: PLoS One. 2013 Oct 29;8(10):e77894. doi: 10.1371/journal.pone.0077894 (PMC3812168; doi:10.1371/journal.pone.0077894)
Supplement: Table S4 — Signalling pathways and identified proteins involved. List of Reactome, KEGG and BioCarta pathways and proteins involved with corresponding p-values. (PDF) [file pone.0077894.s006.pdf]

Supplementary Table S4 Signaling pathways and identified proteins involved

Cluster[a)]	Pathway	Category:Id[b)]	Protein (SSP)[c)]	P adj.[d)]	P-value
4, 14, 15	Metabolism of carbohydrate	Reactome:474	Dihydrolipoyl dehydrogenase (66)<br>α-enolase (96, 103, 104, 105, 106, 110)<br>γ-enolase (107)<br>fructose.bisphosphate aldolase A (134)<br>fructose-bisphosphate aldolase C (132)<br>LDH-B (147, 149)<br>mAspAT (129)<br>Mdh1 (151)<br>phosphoglycerate mutase 1 (184, 187)<br>transaldolase (137)<br>triosephosphate isomerase (193, 194)	6.97E-06	2.32E-07
4, 15	Metabolism of amino acids	Reactome:13	Dihydrolipoyl dehydrogenase (66)<br>mAspAT (129)<br>PA28 α (177)<br>PA28 β (174)<br>proteasome subunit α3 (181)<br>26S proteasome non-ATPase 14 (155)	0.0788	0.027
4, 14, 15	DNA Replication	Reactome:383	MCM7 (6, 19)<br>PA28 α (177)<br>PA28 β (174)<br>PCNA (157)<br>proteasome subunit α3 (181)<br>26S proteasome non-ATPase 14 (155)	0.0497	0.0084
4, 15	Cell Cycle Checkpoints	Reactome:1538	DNA replication licensing factor MCM7 (6, 19)<br>PA28 α (177)<br>PA28 β (174)<br>proteasome subunit α3 (181)<br>26S proteasome non-ATPase 14 (155)	0.077	0.0293
4, 15	Antigen processing and presentation	KEGG:hsa04612	ERp60 (62, 64, 63)<br>GRP-78 (25, 26, 27)<br>HSP90AB (153)<br>PA28 α (177)<br>PA28 β (174)	0.0973	0.0059

Supplementary Table S4 Signaling pathways and identified proteins involved

2R	FAS signaling pathway (CD95)	BioCarta:fasPathway	Lamin-B1 (41)<br>PAK-2 (57)<br>Rho GDI 2 (206)	0.0118	0.0026
7R	Metabolism of carbohydrate	Reactome:474	α-Enolase (103)<br>citrate synthase (118)<br>fructose.bisphosphate aldolase A (134)<br>LDH-B (147, 149)<br>mAspAT (129)<br>Mdh2 (152)<br>phosphoglycerate mutase 1 (184)	0.0017	6.93E-05
7R	Integration of energy metabolism	Reactome:1505	α-Enolase (103)<br>citrate synthase (118)<br>fatty acid synthase (96)<br>fructose.bisphosphate aldolase A (134)<br>Mdh2 (152)<br>phosphoglycerate mutase 1 (184)	0.0425	0.0035
9R	Pyruvate metabolism and TCA cycle	Reactome:1046	Citrate synthase (118)<br>LDH-B (147, 149)<br>Mdh2 (152)	0.00435	0.0012
10R	Glyoxylate and dicarboxylate metabolism	KEGG:hsa630	Citrate synthase (118)<br>Mdh2 (152)	0.0518	0.0059
Ramos	Regulation of actin cytoskeleton	KEGG:hsa4810	β-Actin (115, 114, 112, 121, 127, 122, 162, 173, 198)<br>Arp2/3 (221)<br>cofilin-1 (222)<br>ezrin (21)<br>myosin regulatory 12A (216)<br>PAK-2 (57)	0.2514	0.0149

a) Cluster numbers referring to those in Figures 3 and 4. Ramos corresponds to entire Ramos data set (all proteins in Fig. 4).

b) Category referring to signaling pathway and the identification code in the pathway.

c) Sample spot number (SSP) referring to those in Figure 2 and Table S2.

d) P-value adjusted by the Benjamini multiple test adjustment.
